# Implementation science of Yoga: an integrated approach to health and wellness

**DOI:** 10.3389/fpubh.2026.1757700

**Published:** 2026-07-23

**Authors:** Vinod Srivastava, Swati Kumari Choudhary, J. M. Balamurugan, H. R. Nagendra, Mitali Mukerji, Kalyan Maity, Monika Gautam, Rajvi Mehta, Hemant Bhagat, Anumeha Bhagat, Rima Dada, Hemant Bhargav, Nandi Krishnamurthy Manjunath, Richard R. Fletcher, Alyssa Wostrel, Molly McManus, Susan Steiger Tebb, Om Prakash Katare, Saurabh Kumar, Sanjib Patra, Kashinath Metri, Vikram Pai, H. S. Vadiraja, Aruna Raka, Krishan Kumar, Pramod Kumar Avti, Rakesh Mittal, Neelam Dahiya, Gurmeet Singh, Sumeet Mahajan, Raghavendra M. Rao, Akshay Anand

**Affiliations:** 1 College of Health and Behavioral Sciences, Fort Hays State University, Hays, KS, United States; 2 Interdisciplinary Centre for Swami Vivekananda Studies, Panjab University, Chandigarh, India; 3 Neuroscience Research Lab, Department of Neurology, Postgraduate Institute of Medical Education and Research (PGIMER), Chandigarh, India; 4 Government of Punjab, Punjab, India; 5 Swami Vivekananda Yoga Anusandhana Samsthana (S-VYASA) University, Bengaluru, India; 6 Department of Bioscience and Bioengineering, Indian Institute of Technology Jodhpur, Jodhpur, India; 7 Central Council for Research in Yoga and Naturopathy (CCRYN)–Collaborative Centre for Mind Body Intervention through Yoga, Postgraduate Institute of Medical Education and Research (PGIMER), Chandigarh, India; 8 BKS Iyengar Yogashraya, Mumbai, India; 9 Department of Anaesthesia and Intensive Care, Postgraduate Institute of Medical Education and Research (PGIMER), Chandigarh, India; 10 Department of Physiology, Government Medical College and Hospital-32, Chandigarh, India; 11 Department of Anatomy, All India Institute of Medical Sciences, New Delhi, India; 12 Department of Integrative Medicine, National Institute of Mental Health and Neurosciences (NIMHANS), Bengaluru, India; 13 Massachusetts Institute of Technology, Cambridge, MA, United States; 14 International Association of Yoga Therapy, Little Rock, AR, United States; 15 University Institute of Pharmaceutical Science, Panjab University, Chandigarh, India; 16 Department of Yoga, Central University of Rajasthan, Rajasthan, India; 17 Ayurveda, Yoga and Naturopathy, Unani, Siddha, and Homeopathy (AYUSH), All India Institute of Medical Sciences, Raipur, India; 18 Central Council for Research in Yoga and Naturopathy (CCRYN), Karnataka, India; 19 Department of Translational and Regenerative Medicine, Postgraduate Institute of Medical Education and Research (PGIMER), Chandigarh, India; 20 Department of Psychiatry, Postgraduate Institute of Medical Education and Research (PGIMER), Chandigarh, India; 21 Department of Biophysics, Postgraduate Institute of Medical Education and Research (PGIMER), Chandigarh, India; 22 Skylite Innovations Private Limited, Solan, Himachal Pradesh, India; 23 Department of Cardiology, Postgraduate Institute of Medical Education and Research (PGIMER), Chandigarh, India; 24 Department of Physical Education, Panjab University, Chandigarh, India; 25 School of Chemistry & Chemical Engineering, University of Southampton, Southampton, United Kingdom; 26 Central Council for Research in Yoga and Naturopathy, New Delhi, India

**Keywords:** Yoga, implementation science, integrative medicine, healthcare systems, evidence-based practice, quality management, health policy, stakeholder engagement

## Abstract

This article is a structured narrative review that synthesizes empirical findings and expert consensus and provides policy guidance to outline implementation pathways for integrating Yoga into mainstream healthcare systems, with India as a case example. Yoga is an ancient health practice traditionally used as a holistic approach to health management and wellbeing. However, the acceptance of Yoga as an evidence-based practice in the modern healthcare delivery requires systematic reassessment for its implementation within the healthcare system to make healthcare more affordable, improve prevention, rehabilitation, and resilience thus reduce strain on medical resources. In this review, we have organized the literature review and practical insights around three core themes: stakeholder configurations and governance, economic and quality management, and implementation strategies and evaluation to develop an actionable framework for an optimal care system responsive to patients' needs and adaptable to different community settings. This article has incorporated peer-reviewed studies on the effectiveness of Yoga, its implementation in mainstream healthcare, along with relevant publicly available policy, accreditation, and practice standard sources, to emphasize constructs of implementation science and evidence-based practice. The review identifies a know-do gap between growing evidence for Yoga and its system-level adoption, proposing a staged framework that integrates stakeholder engagement, economic evaluation, quality management, and monitoring to support evidence-based implementation. We propose a comprehensive, context-sensitive model that combines traditional wisdom with contemporary behavioral, clinical, and systems-based approaches. By embedding Yoga within an implementation science framework, we advocate for its role as a mainstream, evidence-informed system. Adoption of Yoga as a therapeutic and preventive modality can be accelerated through standardized protocols, transparent evaluation and regulation, and tripartite governance among state actors, healthcare institutions, and Yoga professionals; however, the feasibility hinges on context-sensitive financial support, stakeholder management, and workforce development.

## 1 Introduction

Yoga is a science of mind-body practice, rooted in ancient Indian health practices, that uses a holistic approach, including non-theistic spiritual and psychological pursuits for physical health, psychological, and spiritual wellbeing. It is an ancient Indian discipline of health, wellness, and lifestyle. Yoga, as a mind-body discipline, incorporates physical postures (asanas), breathing exercises (pranayama), and meditation (dhyana) to harmonize individual consciousness with universal consciousness, balancing mind-body correspondence (1, 2). A growing body of empirical literature has examined Yoga-based interventions across a range of health conditions, including chronic pain, mental health disorders, cardiometabolic disease, and other non-communicable conditions (3–6).

Despite this expanding evidence base, Yoga has not been systematically integrated into mainstream healthcare delivery structures. Much of the existing literature emphasizes clinical efficacy and outcomes, while comparatively limited attention has been given to how Yoga can be operationalized, governed, financed, regulated, and evaluated within real-world health systems. Prior work typically addresses clinical efficacy or broad advocacy for traditional medicine, and few sources explicitly integrate stakeholder mapping, cost, and quality considerations, and policy alignment into a single implementation schema focused on Yoga.

Implementation science provides a structured analytical framework for examining this know–do gap by focusing on the strategies, mechanisms, and contextual factors that influence the adoption, fidelity, sustainability, and scale-up of evidence-based practices across healthcare settings. From this perspective, the central challenge for Yoga is not solely whether it is effective, but how it can be responsibly integrated into institutional workflows while maintaining quality, safety, accountability, and cultural integrity.

This structured narrative review examines the implementation science of Yoga, using India as a case example while drawing on relevant international literature. The review focuses on three implementation-relevant domains: stakeholder configurations and governance structures, economic and quality management mechanisms, and implementation strategies and evaluation approaches that shape the feasibility, adoption, and sustainability of Yoga within healthcare systems.

### 1.1 Current insights and broader considerations in implementation science

Traditional and complementary medicine continues to be used widely, yet many practices remain variably regulated, inconsistently standardized, and unevenly integrated within formal healthcare delivery systems (7, 8). Within this landscape, Yoga stands out because it has a growing empirical literature examining clinical outcomes across multiple conditions.

Evidence for Yoga has expanded across multiple conditions; however, system-level integration remains limited because implementation determinants are not routinely addressed. Key barriers include unclear governance and accountability, inconsistent standardization across education, training, and clinical delivery, variable workforce training and credentialing pathways, limited reimbursement mechanisms, and weak alignment with institutional quality management and evaluation systems. Implementation science provides a framework to examine these determinants through organizational readiness, stakeholder alignment, fidelity and adaptation processes, and sustainability conditions that shape routine adoption in real-world care. Furthermore, integration occurs within complex care pathways, and effective implementation typically requires interprofessional coordination and clearly defined roles, referral pathways, and communication processes across clinical and supportive services. In this review, peer-reviewed literature is synthesized and interpreted using an implementation science lens; CCRYN–PGIMER stakeholder deliberations are used only as contextual input and are not treated as evidence, as described in Section 2.

## 2 Methods

### 2.1 Review design

This study was conducted as a structured narrative review informed by scoping review principles, with an explicit focus on implementation science rather than clinical efficacy alone. The objective was to map and synthesize evidence relevant to system-level adoption, delivery, and sustainability of Yoga-based interventions across real-world settings.

### 2.2 Data source and search strategy

A structured search was conducted in PubMed. The final PubMed query was executed on 14 April 2026 and restricted to human studies published in English, yielding 143 records exported for screening and documentation (Supplementary Table S1). Search terms were developed to capture Yoga and implementation-relevant constructs and included combinations of terms related to implementation, integration, feasibility, acceptability, fidelity, adoption, policy, governance, reimbursement, and service delivery.

### 2.3 Eligibility and screening

Records were screened to determine relevance to implementation of Yoga within healthcare, educational, and community or justice settings. Because the aim was to synthesize implementation-relevant evidence, priority was given to sources that explicitly addressed implementation constructs (for example, feasibility, acceptability, barriers or facilitators, fidelity, adoption, integration into existing systems, policy or governance, guideline development, reimbursement or insurance) rather than reporting clinical outcomes alone.

### 2.4 Source selection and data charting

To provide a comparative presentation of implementation-relevant evidence, Table 1 was constructed as a descriptive summary of an implementation-explicit subset of the PubMed retrieval. Records were included in Table 1 when the title clearly signaled an implementation orientation (for example, feasibility, acceptability, fidelity, barriers or facilitators, implementation, integration, policy or governance, guidelines, reimbursement or insurance, and surveys of practice or adoption), resulting in 59 records (Table 1). For each record in Table 1, publication type or study design and the primary implementation focus were coded descriptively using title cues and PubMed record fields; setting was coded as healthcare, education, justice/carceral, or mixed/other based on title and indexing cues. Given heterogeneity in designs and contexts, Table 1 is descriptive and does not imply quantitative pooling of outcomes.

**Table 1:** Implementation-explicit evidence (*n* = 59) from the retrieved corpus of yoga studies (*n* = 143).

First author (Year)	Study title	Setting	Study design/Type	Primary implementation focus	Implementation relevance note (title- and keyword-based)
Algül G (2026)	Integration of music, yoga and massage: investigation of their effects on preschool children's social-emotional development and quality of life	Education	Interventional study	System integration	Addressed integration of yoga into existing education systems or programs
Ghosh M (2025)	Scope and role of integrating Yoga in homeopathic medical education: an explorative review	Education	Narrative/exploratory review	System integration	Addressed integration of yoga into existing education systems or programs
Wimmer L (2019)	Improving emotion regulation and mood in teacher trainees: effectiveness of two mindfulness trainings	Education	Other (not specified in title)	Program delivery/adherence	Examined program delivery and adherence outcomes relevant to yoga implementation
Butzer B (2015)	School-based Yoga programs in the United States: a Survey	Education	Survey/cross-sectional study	Practitioner/patient survey	Surveyed practitioners or patients on yoga attitudes, knowledge, and practices
Tolbaños-Roche L (2026)	Integrated comprehensive care in short-stay psychiatric units: a yoga-based adjunctive intervention and its association with wellbeing and patient satisfaction	Healthcare	Interventional study	System integration	Addressed integration of yoga into existing healthcare systems or programs
Mona M (2025)	Development, validation, and feasibility of Yoga intervention module for optimizing safe use of screen time: a pilot study	Healthcare	Feasibility/pilot study	Feasibility	Assessed feasibility of yoga delivery in healthcare settings
Chopin SM (2025)	Feasibility of a virtual adaptation of a Yoga intervention for veterans with chronic pain and posttraumatic stress disorder	Healthcare	Feasibility/pilot study	Feasibility	Assessed feasibility of yoga delivery in healthcare settings
Ward L (2025)	Perceptions and experiences of chair-based yoga by older adults with multimorbidity—a qualitative process evaluation of the Gentle Years Yoga randomized controlled trial	Healthcare	Qualitative study/process evaluation	Process evaluation	Conducted qualitative process evaluation of yoga program delivery
Santarossa S (2025)	Equity-focused barriers and facilitators to implementing a prenatal yoga intervention in a healthcare system: patient and provider perspectives	Healthcare	Qualitative study/process evaluation	Barriers and facilitators	Identified barriers and facilitators to yoga implementation in healthcare settings
Loy M (2024)	Implementation of Virtual Yoga Shared Medical Appointment (VYSMA) pilot at an academic medical center within a mixed-diagnosis oncology population	Healthcare	Feasibility/pilot study	Implementation evaluation	Examined implementation processes and outcomes for yoga in healthcare settings
Zaccari B (2024)	Trauma center trauma-sensitive Yoga vs. cognitive processing therapy for women veterans with PTSD who experienced military sexual trauma: a feasibility study	Healthcare	Feasibility/pilot study	Feasibility	Assessed feasibility of yoga delivery in healthcare settings
Hall S (2024)	Implementation of a clinic-based Yoga program for chronic pain	Healthcare	Implementation study/report	Implementation evaluation	Examined implementation processes and outcomes for yoga in healthcare settings
McCurdy BH (2024)	Program evaluation of a school-based mental health and wellness curriculum featuring yoga and mindfulness	Healthcare	Program/process evaluation	Program evaluation	Evaluated a yoga-based wellness or mental health program in practice
Kligler B (2023)	What we have learned about the implementation of whole health in the veterans administration	Healthcare	Implementation study/report	Implementation evaluation	Examined implementation processes and outcomes for yoga in healthcare settings
Smit C (2023)	Recommending yoga for health: a survey of perceptions among healthcare practitioners in the UK	Healthcare	Survey/cross-sectional study	Practitioner/patient survey	Surveyed practitioners or patients on yoga attitudes, knowledge, and practices
Kaur S (2022)	Evaluation of an integrated yoga program in patients with inflammatory bowel disease: a pilot study	Healthcare	Feasibility/pilot study	System integration	Addressed integration of yoga into existing healthcare systems or programs
Ahmadi E (2022)	Assessing the acceptability of yoga among patients with and without chronic pain enrolled in a licensed opioid treatment program	Healthcare	Other (not specified in title)	Acceptability	Evaluated acceptability of yoga among the target population
Chaitanya Putchavayala K (2022)	Development, content validation, and feasibility of Yoga module for smartphone addiction	Healthcare	Feasibility/pilot study	Feasibility	Assessed feasibility of yoga delivery in healthcare settings
Heeter C (2021)	Feasibility, acceptability, and outcomes of a yoga-based meditation intervention for hospice professionals to combat burnout	Healthcare	Feasibility/pilot study	Feasibility and acceptability	Assessed feasibility and acceptability of yoga delivery in the study context
Dhungana RR (2021)	Yoga for hypertensive patients: a study on barriers and facilitators of its implementation in primary care	Healthcare	Implementation study/report	Barriers and facilitators	Identified barriers and facilitators to yoga implementation in healthcare settings
Anderson BJ (2021)	Barriers and facilitators to implementing bundled acupuncture and yoga therapy to treat chronic pain in community healthcare settings: a feasibility pilot	Healthcare	Feasibility/pilot study	Barriers and facilitators	Identified barriers and facilitators to yoga implementation in healthcare settings
Salwa H (2020)	Raising burden of non-communicable diseases: importance of integrating Yoga and Naturopathy at primary care level	Healthcare	Other (not specified in title)	System integration	Addressed integration of yoga into existing healthcare systems or programs
Sharma KNS (2020)	Integrated yoga practice in cardiac rehabilitation program: a randomized control trial	Healthcare	Randomized controlled trial	System integration	Addressed integration of yoga into existing healthcare systems or programs
Lai KSP (2020)	Breath Regulation and yogic Exercise An online Therapy for calm and Happiness (BREATH) for frontline hospital and long-term care home staff managing the COVID-19 pandemic: a structured summary of a study protocol for a feasibility study for a randomized controlled trial	Healthcare	Study protocol (randomized trial)	Feasibility	Protocol describes feasibility and implementation outcomes to be assessed in the study context
Flehr A (2019)	#MindinBody - feasibility of vigorous exercise (Bikram yoga vs. high intensity interval training) to improve persistent pain in women with a history of trauma: a pilot randomized control trial	Healthcare	Feasibility/pilot study	Feasibility	Assessed feasibility of yoga delivery in healthcare settings
Taylor MJ (2017)	Implementation of yoga therapy into U.S. Healthcare Systems	Healthcare	Implementation study/report	Implementation evaluation	Examined implementation processes and outcomes for yoga in healthcare settings
Waddington EA (2017)	Staff perspectives regarding the implementation of a yoga intervention with chronic pain self-management in a clinical setting	Healthcare	Qualitative study/process evaluation	Implementation evaluation	Examined implementation processes and outcomes for yoga in healthcare settings
Brems C (2015)	Improving access to yoga: barriers to and motivators for practice among health professions students	Healthcare	Other (not specified in title)	Barriers to adoption	Identified barriers to yoga adoption or practice in healthcare contexts
Slocum-Gori S (2013)	Investigating the perceived feasibility of integrative medicine in a conventional oncology setting: yoga therapy as a treatment for breast cancer survivors	Healthcare	Feasibility/pilot study	System integration	Addressed integration of yoga into existing healthcare systems or programs
Krupp K (2025)	Policy implications of integrating yoga for healthy aging: a stakeholder analysis in India	Other/Mixed	Policy analysis/commentary	Policy and governance	Analyzed policy implications and governance frameworks for yoga integration
Allene C (2025)	Yoga as a pre-treatment of EMDR to treat childhood abuse-related PTSD: feasibility and pilot study	Other/Mixed	Feasibility/pilot study	Feasibility	Assessed feasibility of yoga delivery in mixed settings
Karim FS (2025)	Knowledge, attitude, practice, and perceived barriers to antenatal yoga among obstetricians and gynecologists in Bangladesh: a cross-sectional survey	Other/Mixed	Survey/cross-sectional study	Barriers to adoption	Identified barriers to yoga adoption or practice in mixed contexts
Pradhan N (2025)	Understanding the feasibility of yoga and compassion meditation for stress management in mothers of children with intellectual disabilities in Nepal: a qualitative exploration	Other/Mixed	Qualitative study/process evaluation	Feasibility	Assessed feasibility of yoga delivery in mixed settings
Irfan N (2025)	Integrating AI predictive analytics with naturopathic and yoga-based interventions in a data-driven preventive model to improve maternal mental health and pregnancy outcomes	Other/Mixed	Interventional study	System integration	Addressed integration of yoga into existing mixed systems or programs
Saint K (2024)	Assessing fidelity in yoga interventions for chemotherapy-induced peripheral neuropathy: decision-making to enhance protocol quality adherence	Other/Mixed	Interventional study	Intervention fidelity	Examined fidelity assessment strategies to ensure yoga protocol quality
Krishnan A (2024)	Integrative treatment for substance use disorders: improving outcomes through evidence-based practice of yoga-derived breathwork and meditation interval training) to improve persistent pain in women with a history of trauma: a pilot randomized control trial	Other/Mixed	Other (not specified in title)	System integration	Addressed integration of yoga into existing mixed systems or programs
Wehrmann R (2024)	Peer support groups integrated with trauma-sensitive yoga for women survivors of sexual violence: a feasibility study and qualitative examination	Other/Mixed	Qualitative study/process evaluation	System integration	Addressed integration of yoga into existing mixed systems or programs
Telles S (2023)	Patients' choices for yoga therapy: an exploratory cross-sectional, convenience-sampling survey from India	Other/Mixed	Survey/cross-sectional study	Practitioner/patient survey	Surveyed practitioners or patients on yoga attitudes, knowledge, and practices
Nilkantham S (2023)	Knowledge, attitude, and practice of yoga for thyroid dysfunction: a cross-sectional survey using a tableau approach	Other/Mixed	Survey/cross-sectional study	Practitioner/patient survey	Surveyed practitioners or patients on yoga attitudes, knowledge, and practices
Moonaz S (2023)	Development and implementation of a flexible yoga therapy protocol in the Group Acupuncture Therapy and Modified Yoga (GAPYOGA) pilot study	Other/Mixed	Feasibility/pilot study	Implementation evaluation	Examined implementation processes and outcomes for yoga in mixed settings
Kwok JYY (2023)	A randomized controlled trial on the effects and acceptability of individual mindfulness techniques —meditation and yoga—on anxiety and depression in people with Parkinson's disease: a study protocol	Other/Mixed	Study protocol (randomized trial)	Acceptability	Evaluated acceptability of yoga among the target population
Ward L (2022)	Development of the CLARIFY (CheckList standardizing the Reporting of Interventions For Yoga) guidelines: a Delphi study	Other/Mixed	Guideline/Delphi consensus	Reporting guidelines	Developed or elaborated consensus-based guidelines for standardized yoga reporting
O'Shea M (2022)	Integration of hatha yoga and evidence-based psychological treatments for common mental disorders: an evidence map	Other/Mixed	Systematic review/evidence synthesis	System integration	Addressed integration of yoga into existing mixed systems or programs
Jakicic JM (2021)	Feasibility of integration of yoga in a behavioral weight-loss intervention: a randomized trial	Other/Mixed	Feasibility/pilot study	System integration	Addressed integration of yoga into existing mixed systems or programs
Sohl SJ (2021)	Ensuring Yoga Intervention Fidelity in a Randomized Preference Trial for the Treatment of Worry in Older Adults	Other/Mixed	Randomized controlled trial	Intervention fidelity	Examined fidelity assessment strategies to ensure yoga protocol quality
Brosnan P (2021)	Acceptability and feasibility of the online delivery of hatha yoga: a systematic review of the literature	Other/Mixed	Systematic review/evidence synthesis	Feasibility and acceptability	Assessed feasibility and acceptability of yoga delivery in the study context
Moonaz S (2021)	CLARIFY 2021: explanation and elaboration of the Delphi-based guidelines for the reporting of yoga research interval training) to improve persistent pain in women with a history of trauma: a pilot randomized control trial	Other/Mixed	Guideline/Delphi consensus	Reporting guidelines	Developed or elaborated consensus-based guidelines for standardized yoga reporting
Gorvine MM (2021)	An exploratory study of the acceptability and feasibility of yoga among women in substance use disorder recovery	Other/Mixed	Feasibility/pilot study	Feasibility and acceptability	Assessed feasibility and acceptability of yoga delivery in the study context
Huberty J (2020)	Online yoga to reduce post-traumatic stress in women who have experienced stillbirth: a randomized control feasibility trial	Other/Mixed	Feasibility/pilot study	Feasibility	Assessed feasibility of yoga delivery in mixed settings
Nicotera N (2020)	The influence of Trauma-Informed Yoga (TIY) on emotion regulation and skilled awareness in sexual assault survivors	Other/Mixed	Other (not specified in title)	Program delivery/adherence	Examined program delivery and adherence outcomes relevant to yoga implementation
Law H (2020)	Recruitment, retention, and adherence in a randomized feasibility trial of mindfulness-based stress reduction for patients with migraine	Other/Mixed	Feasibility/pilot study	Feasibility	Assessed feasibility of yoga delivery in mixed settings
Donnelly KZ (2017)	The feasibility and impact of a yoga pilot programme on the quality-of-life of adults with acquired brain injury	Other/Mixed	Feasibility/pilot study	Feasibility	Assessed feasibility of yoga delivery in mixed settings
Ross A (2016)	The implementation of patient-reported outcome measures in yoga therapy	Other/Mixed	Implementation study/report	Implementation evaluation	Examined implementation processes and outcomes for yoga in mixed settings
Sheffield KM (2016)	Efficacy, feasibility, and acceptability of perinatal yoga on women's mental health and wellbeing: a systematic literature review	Other/Mixed	Systematic review/evidence synthesis	Feasibility and acceptability	Assessed feasibility and acceptability of yoga delivery in the study context
Vinchurkar SA (2015)	Integrating yoga therapy in the management of urinary incontinence: a case report	Other/Mixed	Case report	System integration	Addressed integration of yoga into existing mixed systems or programs
Albert S (2015)	Is “mainstreaming AYUSH” the right policy for Meghalaya, northeast India?	Other/Mixed	Policy analysis/commentary	Policy and governance	Analyzed policy implications and governance frameworks for yoga integration
Caffrey MK (2014)	Evidence builds on yoga, but no reimbursement yet	Other/Mixed	Other (not specified in title)	Reimbursement/financing	Reviewed reimbursement and insurance landscape for yoga-based services
Bonura KB (2011)	The psychological benefits of yoga practice for older adults: evidence and guidelines	Other/Mixed	Other (not specified in title)	Reporting guidelines	Developed or elaborated consensus-based guidelines for standardized yoga reporting
Lange B (2010)	The yoga mat as common ground: policy from a holistic perspective	Other/Mixed	Policy analysis/commentary	Policy and governance	Analyzed policy implications and governance frameworks for yoga integration

Records were selected from the full PubMed search (n = 143) on the basis of titles explicitly signaling implementation-relevant constructs (implementation, feasibility, acceptability, fidelity, barriers or facilitators, policy or governance, guidelines, reimbursement or insurance, integration, and surveys), spanning healthcare, educational, and other/mixed settings. Study design/type and primary implementation focus were coded descriptively using title cues and PubMed record fields (including keywords where available). The complete retrieved corpus is provided in Supplementary Table S1 (n = 143). This table is descriptive and does not imply quantitative pooling of outcomes. Study-specific findings are synthesized narratively in the Results because implementation outcomes and reporting formats vary across studies.

### 2.5 Synthesis approach

Evidence was synthesized narratively using thematic mapping across three implementation domains: (1) stakeholder configurations and governance structures; (2) economic and quality-management mechanisms; and (3) implementation strategies and evaluation approaches. Themes were developed iteratively by grouping sources according to their implementation focus and the domain(s) they informed.

### 2.6 Use of structured expert deliberations

The CCRYN–PGIMER Mind–Body Intervention: Emerging Status Workshop and Expert Meetings (2024–2026) convened multidisciplinary stakeholders, including academicians, clinicians, Yoga therapists, healthcare administrators, policymakers, researchers, students, International Association of Yoga Therapists (IAYT) representatives, and patient representatives. The author group participated in these meetings and used them only to contextualize feasibility considerations and real-world constraints described in the published literature and to support the interpretation of implementation barriers and facilitators. These deliberations were not treated as an empirical dataset, were not analyzed as study evidence, and did not contribute independent findings; recommendations that extend beyond the published implementation literature are explicitly labeled as consensus-based or exploratory.

### 2.7 Methodological considerations

This review was designed as a structured narrative synthesis informed by scoping principles and drew on heterogeneous sources; we did not undertake a formal assessment of potential bias across all included materials. Instead, interpretation emphasized transparency of source type, clarity of implementation relevance, and convergence of themes across settings. Following this approach, we included articles from PubMed and created Table 1 classification.

## 3 Results

### 3.1 Evidence identification and characteristics

The PubMed search (human studies, English) yielded 143 records (Supplementary Table S1). From this corpus, an implementation-explicit subset of 59 records was identified based on title cues and abstracts, indicating implementation relevance (e.g., feasibility, acceptability, fidelity, barriers or facilitators, policy or governance, guideline development, reimbursement or insurance, integration, and surveys). Table 1 provides a descriptive, comparative summary of this subset by setting and title-coded study design/type, along with implementation-relevance notes. Key implementation findings across these studies are synthesized narratively in Sections 3.2 to 3.3 due to heterogeneity in designs, outcomes, and reporting, while Table 1 supports comparisons of settings, study types, and implementation focus. The implementation-explicit evidence base is heterogeneous in design and context, with substantial representation of feasibility/pilot work, qualitative or process evaluation studies, and surveys, while fewer sources address financing, reimbursement, and system-level governance mechanisms in an evaluative manner. The results are organized and presented in a narrative format across the three predetermined implementation domains.

To provide a comparative presentation of the implementation-explicit evidence base, Table 1 summarizes the PubMed-derived implementation subset (*n* = 59) by setting and title-coded study design/type, with implementation relevance notes.

In addition to the published literature, CCRYN–PGIMER stakeholder deliberations were used only as contextual input; these perspectives are interpretive and do not constitute empirical findings.

### 3.2 Integration of literature evidence with contextual stakeholder input

The explicit articles on implementation include a limited number of records that directly address governance, policy alignment, and system integration for Yoga within the mainstream healthcare system and its related frameworks. In Table 1, policy- and governance-oriented items are represented primarily by titles framed as policy analyses, stakeholder analyses, or system integration discussions (e.g., policy implications and stakeholder analysis; policy perspective; and AYUSH mainstreaming discourse). These sources collectively indicate that implementation challenges extend beyond clinical delivery and include questions of institutional ownership, accountability, and alignment with existing service structures and decision pathways (Table 1).

In addition to explicit policy pieces, reporting and standardization-oriented records represent governance-adjacent mechanisms because they focus on specifying what should be reported and how Yoga interventions should be described in research and practice settings. Such records signal that governance in this area is closely linked to standardization and transparency, including specification of intervention components, documentation practices, and the conditions under which adaptation is acceptable (Table 1).

The articles in Table 1 suggest that governance and stakeholder architecture are less frequently addressed than feasibility and acceptability studies. This pattern suggests that the evidence base is stronger for local delivery questions than for system-level questions, such as governance arrangements, credentialing authority, institutional accountability, and formal integration within health service referral and oversight structures (Table 1; Supplementary Table S1).

Findings from the literature synthesis were examined alongside CCRYN–PGIMER stakeholder deliberations to corroborate feasibility considerations and to inform pragmatic sequencing of implementation strategies; these inputs were not treated as independent evidence.

### 3.3 Emergence of thematic domains

Economic and quality management mechanisms are relatively underrepresented in the implementation science of yoga, as shown in the subset of retrieved articles. In Table 1, the most relevant economic indicator is a record focused on reimbursement, highlighting the importance of financing as a factor in the implementation of yoga. However, the limited number of titles addressing financing suggests that reimbursement pathways and cost structures remain poorly defined in the existing implementation literature.

Quality management is more often represented through fidelity- and standardization-oriented records. Table 1 includes fidelity-focused items and guideline-oriented items that point to protocol quality adherence and standardized reporting, respectively. Together, these sources indicate that quality management concerns in Yoga implementation have been addressed primarily through the lens of protocol fidelity, documentation, and reporting standards rather than through formal institutional quality assurance systems, accreditation, or routinely monitored performance indicators (Table 1).

This pattern is implementation-relevant because financing and quality management mechanisms are central to sustainability and routinization within real-world systems. The limited representation of reimbursement and institutional quality management in Table 1 suggests that these determinants are less frequently examined than early-stage feasibility, acceptability, and localized delivery studies (Table 1; Supplementary Table S1).

Thematic domains were developed through an iterative, deductive, and inductive process of synthesizing evidence and information in the context of implementation science relevance. Initial domains were informed by established constructs in implementation science, including governance, stakeholder engagement, economic evaluation, quality management, intervention fidelity, adaptation, and sustainability. These domains were subsequently refined through repeated examination of the included literature and expert deliberation outputs, allowing consolidation of overlapping concepts and clarification of domain boundaries.

Across both evidence sources, three overarching thematic domains consistently recurred:

1. Stakeholder configurations and governance structures necessary for legitimizing, coordinating, and sustaining Yoga within healthcare systems.
2. Economic and quality management mechanisms required to demonstrate value, ensure accountability, and support institutional adoption.
3. Systematic implementation strategies and evaluation processes that balance standardization with contextual adaptation while maintaining clinical, organizational, and cultural fidelity.

Thematic synthesis, along with evidence appraisal, was conducted at the level of domains rather than individual studies. The strength of evidence assigned to each thematic domain reflects convergence and consistency of implementation-relevant findings across sources, consideration of methodological rigor where applicable, and corroboration by real-world practice insights; however, where evidence was inconsistent or not reliably available, expert insights were used for interpretive framing and to generate consensus based recommendations, and not to upgrade the evidence strength classification. As a result, evidence supporting thematic domains was classified as robust, moderate, or emerging, to provide an analytic foundation for interpretation in Section 4.

## 4 Discussion

The implementation-explicit literature indicates that Yoga integration is most commonly addressed at the level of feasibility, acceptability, and localized delivery adaptations, rather than through system-level implementation infrastructures. Across settings, studies frequently emphasize early-stage implementation questions (for example, whether delivery is feasible, acceptable, and deliverable with adequate adherence), with fewer sources addressing governance accountability, reimbursement mechanisms, workforce credentialing, and alignment with institutional quality management systems. This pattern suggests that the primary barrier to scale-up is not only the lack of evidence of clinical benefit but also the absence of routinized implementation supports, including clearly defined roles, referral pathways, and evaluation and quality assurance structures.

### 4.1 Implementation science of Yoga

Implementation science is a relatively new discipline concerned with systematically addressing the long-standing “know–do gap” by identifying and mitigating obstacles that prevent the uptake of empirically supported interventions and evidence-based practices. Its central aim is to improve population health and reduce disparities by ensuring that effective interventions are not only developed but are also adopted, implemented, and sustained across real-world settings. Within this context, implementation science provides a structured and conceptually rigorous lens through which the integration of traditional medicine modalities into contemporary healthcare systems can be examined and operationalized.

Although there is an expanding literature on the effectiveness of Yoga, portraying a comprehensive picture of Yoga's therapeutic potential, there remains a notable absence of clear strategies or formalized implementation pathways for integrating Yoga with existing healthcare modalities and for ensuring condition-specific, co-managed treatment protocols. However, as healthcare systems around the world move toward prevention strategies, the importance of Yoga is increasing. Consequently, in the West, insurance companies' interest in Yoga is growing somewhat due to its preventive and therapeutic qualities, which can reduce the potential financial burden on insurers. However, insurance coverage for Yoga remains rare, mostly confined to specific cases like cardiac rehabilitation programs offered by select institutions. In India, despite Yoga's cultural embeddedness and the substantial scientific evidence supporting its health benefits, healthcare professionals have not yet widely accepted Yoga as a therapeutic modality within formal care pathways. These divergent realities underscore an urgent need to raise awareness among healthcare professionals, policymakers, insurance bodies, and the general population about the potential benefits of Yoga and to develop structured, evidence-based approaches for its systematic integration into healthcare systems.

A fundamental requirement for integrating Yoga-based therapeutic protocols into healthcare systems is the establishment of a dedicated regulatory approval pathway, analogous to the Drug Controller General of India (DCGI) process for drugs, biologics, and medical devices. Standardization efforts undertaken by AYUSH and other bodies provide important scaffolding for practice, yet they do not constitute regulatory evaluation. For therapeutic Yoga protocols to be accepted within formal medical pathways, a nodal regulatory authority must assess protocol safety, therapeutic rationale, fidelity requirements, and evidentiary support. This regulatory review must precede implementation as it forms the basis of institutional trust, clinical acceptance, and system-wide adoption.

Thematic domains are explicitly mapped, linkages across constructs are made visible, and statements regarding evidence strength are fully integrated into the analysis. This approach provides a clear, conceptually grounded foundation for subsequent stakeholder analysis and supports the development of an implementation framework aligned with evidence-based practice and contemporary implementation science. This analysis illustrates the effective integration of Yoga into healthcare systems requiring continuous alignment of diverse stakeholder interests, proactive management of conflicts and power differentials, and strategic engagement with public opinion and emerging technologies, ensuring that implementation efforts remain contextually responsive, evidence-aligned, and resilient to systemic challenges.

### 4.2 Thematic analysis and findings

Comparative analysis suggests that institutions will be able to integrate evidence-based Yoga protocols more successfully and meaningfully when advisory stakeholder boards are established at both institutional and state levels and operate with transparent terms, including the absence of conflicts of interest, defined term limits, and interdisciplinary membership well versed in integrative health frameworks, with clearly assigned responsibilities for systematic protocol standardization based on emerging evidence, rigorous evaluation and approval, and ongoing oversight to ensure fidelity, accountability, and coordination across implementation settings. The strength of evidence supporting multi-stakeholder governance in health implementation is moderate, whereas the evidence specific to Yoga-focused boards with defined quality mandates remains emerging. Thus, stakeholder alignment is foundational for implementation feasibility, and effective communication requires bidirectional feedback and staged public messaging to build trust, support transparency, and strengthen social capital.

Economic analyses, including cost-benefit, cost-effectiveness, and cost-utility assessments, are essential for motivating payers, administrators, and policymakers, particularly when introducing new workforce requirements, risk-management systems, and organizational responsibilities. These assessments help determine whether Yoga-based interventions provide measurable value relative to their implementation costs and can guide decisions regarding reimbursement, resource allocation, and program sustainability. The strength of evidence supporting the cost-effectiveness of Yoga for specific clinical indications and for the role of quality systems in health intervention delivery is moderate, while evidence for comprehensive cost-utility analyses within integrative healthcare models is emerging. The successful integration and implementation of Yoga are closely linked to a strong quality management infrastructure capable of monitoring fidelity of approved protocols, tracking outcomes, and maintaining auditable processes. Without such systems, claims of cost savings or efficiency improvements cannot be substantiated, and implementation efforts risk becoming unsustainable or inconsistent across settings. As a result, it is reasonable to conclude that financing strategies must align with quality-assurance structures and robust data systems to ensure verifiability, institutional accountability, and long-term risk management, thereby enhancing the feasibility and sustainability of integrating Yoga into healthcare systems.

The review findings endorse staged implementation of Yoga, staff training, and ongoing supervision with adaptation based on stakeholder feedback. These foundational components remain essential and are retained. Building on these elements, the implementation of Yoga-based programs can be strengthened by aligning strategies with established constructs in implementation science, including organizational readiness, fidelity to standardized protocols, structured adaptation processes, and long-term sustainability planning. In keeping with these principles, phased demonstration projects with clearly defined stop-go-adapt criteria are recommended. Such pilots allow for systematic testing of feasibility and acceptability, followed by expansion only when predefined benchmarks are met. The strength of evidence supporting phased pilots and iterative adaptation within broader implementation science is robust, while evidence specific to Yoga-related pathways in tertiary care settings appears moderate. The successful integration of Yoga into healthcare may depend on deliberate piloting, structured learning cycles, and transparent criteria for scaling, all while maintaining both cultural and clinical fidelity to the intervention.

Monitoring and evaluation must incorporate both quantitative and qualitative methods and include regular stakeholder review cycles to ensure that implementation remains responsive to emerging needs and contextual changes. While traditional evaluation metrics remain central to understanding program effectiveness and fidelity, advances in multiomics technologies, digital biomarkers, and distributed diagnostics offer new opportunities for personalized assessment and real-time adherence monitoring. These tools are promising but remain in early stages of development and should serve as adjuncts rather than replacements for established clinical and patient-reported outcomes. As a result, core clinical and functional measures should remain the foundation of program evaluation, with precision tools adopted selectively and strategically where they enhance, rather than displace, evidence-based assessment practices.

### 4.3 Stakeholder analysis

The integration of Yoga into the primary healthcare system is complex and multifaceted. Successful implementation requires significant collaboration and cooperation among diverse stakeholders, including patients and the general population. Each stakeholder group brings unique perspectives, obligations, and motivations that influence the process of integrating Yoga into healthcare and ensuring patient and community wellbeing. Navigating these complexities and obscurities may require all important stakeholders' involvement, prioritization of the needs of involved parties, and understanding their interests and influences (actual and potential influence) to overcome barriers and concerns for the successful integration of Yoga with the healthcare system (9). However, stakeholders must be representatives from different fields, such as education, medical professionals, Yoga practitioners, administration, and most importantly, consumers, to overcome obstacles and facilitate the integration process. A stakeholder mapping was developed by the author group and informed by CCRYN–PGIMER stakeholder deliberations convened during the “Mind-Body Intervention: Emerging Status Workshop and Expert Meetings.” This mapping is presented as contextual, consensus-based input to support interpretation of published implementation evidence and is not presented as empirical study evidence.

#### 4.3.1 Primary stakeholders

##### 4.3.1.1 Medical professionals and researchers

- *Interests:* good treatment outcomes, reduced treatment burden, assessing alternative treatment options, quality instruction, and training.
- *Influence:* patient care, empirical research, awareness creation, communication with patients, families, and professionals, and evidence-based decision-making involving different empirically evidenced and approved Yoga interventions.

##### 4.3.1.2 Patients and larger communities

- *Interests:* accessibility to holistic/complementary healthcare interventions for better and sustainable outcomes and overall wellbeing to maintain better functionality in the community.
- *Influence:* feedback to professionals, programs, treatment modalities/its decision-making process through active participation in informing and influencing their needs, requirements, and priorities, as well as through community engagement.

##### 4.3.1.3 Yoga instructors and practitioners

- *Interests:* promoting Yoga as a therapeutic tool, quality instruction, and standardizing Yoga practice instructions and outcomes measurements.

- *Influence:* designing and delivering Yoga protocols, programs, and training.

##### 4.3.1.4 Clinical and translational researchers

- *Interests:* generating empirical evidence through rigorous study designs, including randomized controlled trials, translational studies, comparative effectiveness studies, and multi-site evaluations of Yoga protocols.
- *Influence:* establishing the evidence base that informs regulatory approval, guiding protocol refinement through empirical data, and ensuring that implementation is grounded in verified therapeutic benefit rather than assumed efficacy. Their work is essential for validating safety, mechanisms of action, effectiveness, and condition-specific indications for Yoga interventions.

#### 4.3.2 Secondary stakeholders

##### 4.3.2.1 Government health departments

- *Interests:* preventive healthcare practice, low healthcare costs, and general wellbeing of the population.
- *Influence:* prioritizing funding for healthcare institutions, research, policy, and programs for an integrated and effective healthcare system.

##### 4.3.2.2 Healthcare institutions

- *Interests:* various effective healthcare services, better outcomes, and patient satisfaction.
- *Influence:* influencing institutional policies, procedures, rules, practices, and resource allocation for evidence-based Yoga practice/protocols/programs.

##### 4.3.2.3 Educational institutions

- *Interests:* providing relevant education, medical services, research, program/curriculum evaluation, and consumer satisfaction.
- *Influence:* relevant, practical, and experiential learning curriculum designs, services, and training programs for healthcare professionals on integrative medicine.

##### 4.3.2.4 Insurance companies

- *Interests:* cost-effective healthcare and preventive measures to decrease insurance claims, offer competitive premiums to its customers and increase companies' financial health.
- *Influence:* integrating preventive Yoga programs in health insurance coverage and specific Yoga interventions for targeted outcomes.

##### 4.3.2.5 Yoga organizations

- *Interests:* promote Yoga practice and its benefits to people.
- *Influence:* provide expertise and collaboration on program design and implementations.

#### 4.3.3 Tertiary stakeholders

##### 4.3.3.1 Media and advocacy groups

- *Interests:* create awareness of Yoga benefits to influence public opinion on integrative healthcare.
- *Influence:* disseminate accurate information to shape public opinion in favor of the benefits of Yoga and advocate for policy support.

##### 4.3.3.2 Technology providers

- *Interests:* develop technological platforms to make Yoga programs accessible, monitoring compliance, effectiveness, and improvement.
- *Influence:* targeted technological solutions, data management of health information, and analytical programs for program and practice evaluations.

##### 4.3.3.3 Research institutions

- *Interests:* create empirical evidence through research and evaluations to advanced scientific understanding of Yoga protocols and their impact on health and wellbeing.
- *Influence:* conduct rigorous research initiatives (RCTs) for outcome measurements, disseminate empirical evidence, and contribute to evidence-based practices involving Yoga.

In the stakeholder analysis in the manuscript, the attention is more directed toward the primary and secondary stakeholders. It is crucial for implementation science to understand the complexities of stakeholders' interests and what influence they may have on the success of any initiatives. However, it is essential to understand the role of media, technological forms, and influencers in ensuring the success of implementation science. Creating favorable public opinion and enduring acceptance by the general public may prove to be determinants of the success of Yoga in the healthcare system, which highlights the importance of other stakeholders' influence. Recognizing and aligning stakeholders' interests, updating stakeholder engagement strategies periodically, and addressing conflicts of interest proactively are essential for navigating the complexities associated with integrating Yoga into mainstream healthcare. It would significantly help to deal with big companies with vested interests, as it may be seen as “disruptive technology,” a term used to show the potential conflict of interests due to Yoga's preventative nature and possible threats to maintenance drugs people use lifelong or as needed. Navigating potential challenges in an informed way may have an additive effect on overcoming barriers and successfully implementing strategies. Furthermore, the cost-benefit analysis could be crucial in assessing the strategic moves and initiatives.

### 4.4 Economic evaluation and quality management implications of Yoga integration

An economic evaluation of Yoga integration should consider cost-benefit analysis, cost-effectiveness analysis, and cost-utility analysis, as each provides distinct information for reimbursement, resource allocation, institutional risk management, and long-term sustainability. While stakeholder interests and influences are crucial for successful integration, economic strategies to manage and reduce healthcare costs cannot be overlooked. Achieving better patient outcomes, maintaining health, and enhancing patient functionality remain core objectives for healthcare establishments and society at large. Cost-benefit analysis is associated with differential assessment of the costs related to different treatment outcomes (10). In contrast, cost-effectiveness analysis is concerned with costs of desired treatment outcomes with minimal associated costs and years of life gained (as applicable to the illness) (11), and cost-utility analysis measures cost associated in terms of treatment outcomes in terms of quality-adjusted life, disability-adjusted life, and longevity preferences (12). Countries with high living costs and expensive medical treatments depend on insurance companies to make treatment affordable and accessible, whereas in countries like India, treating and preventing health issues may be cost-effective. Access to Yoga through private and health institutions in India can vary significantly. Integrating Yoga into healthcare for specific and targeted treatments introduces various complexities. These include the potential patenting of Yoga protocols, which could increase costs, the expenses associated with hiring trained professionals alongside existing staff, and the need for organizational risk management. Additionally, alignment with legal requirements and reasonable associated costs must be considered, along with the independent assessment, intervention, and evaluation of Yoga protocols and their additive effects on treatment outcomes. Implementing Yoga integration will also require licensing revisions and continuing education for professionals with additional costs. Direct treatment outcomes and outputs associated with the integrated system will not succeed without integration with other professionals, such as nurses, psychologists, biologist, and social workers. Such efforts would require sustained networking and communication for better outcomes and depend on institutional cost-bearing abilities to achieve better outcomes. It would be optimal to propose a subcommittee, even an *ad-hoc* committee, with target institutions, to assess and propose a cost-benefit analysis on the integration of Yoga in the healthcare system and propose contextualized guidelines for its operation and expansion in the healthcare system for effective and adequate integration successively.

Effective quality management is pivotal for optimizing clinical decision-making, streamlining processes, enhancing patient outcomes, and ensuring organizational efficiency—all of which are critical for successfully integrating Yoga into healthcare systems. Active participation in streamlining processes could prove the integration of Yoga into healthcare systems and decrease the overall financial burden on institutions, patients, and insurance companies. Clinical decision-making based on evidence-based best practices may not function well without quality managers and their team's engagement and participation in key decision-making processes, such as managerial and financial decisions and collaboration, and using medical and insurance companies' expertise collaboratively to integrate Yoga and decrease overall cost.

The role of quality management systems within health institutions must be addressed and modified for better and more efficient operation of medical systems and the successful integration of Yoga into the healthcare system. Without a robust quality management system, integrating Yoga may appear less cost-effective and daunting. Without a strong quality system, adding Yoga to patient care seems expensive and tough to handle. Teaching methods differ too much, causing uneven results, extra medical needs, or safety problems that drive up costs. A solid system sets clear training rules, regular checks, and result tracking, making Yoga cheaper over time, and simpler for hospitals to adopt by lowering risks and repeat visits.

Several research initiatives within medical institutions can also benefit from collaboration with quality management professionals and utilize good laboratory practices (GLP) methodology to ensure verifiability of results, effective management systems of lab resources, institutional resource utilization, and outcomes evaluation. Involvement of quality management departments will facilitate synergistic integration of all systemic variables to integrate Yoga and optimize the overall process; it can also be achieved in incremental phases —revamping the quality management department, translating professional expertise and requirements into resources available to others at the initial phase, understanding the needs of different departmental medical and adjunct services following evidence-based practice, and outlining collaborative initiatives based on their needs and need for involvement. It will help them be involved strategically with management, services, and available resources to help them with ongoing needs, streamlining processes and resources, and prioritizing initiatives to decrease health costs and evaluate them. A data-driven transparent approach, established communication, systems of cooperation and coordination, and flow of confidential information within systems with accuracy and credibility, auditability, and verifiability would decrease organizational risks and liabilities, increase patients' wellbeing, and streamline financial burden. The use of technological advancements and artificial intelligence can potentially be deployed to aid compliance, verification and systems level understanding at both the therapeutic and economic levels. These economic considerations and quality-management requirements underscore that the successful integration of Yoga into healthcare systems depends on aligning cost analyses with robust institutional quality structures, ensuring that implementation initiatives remain financially justified, operationally feasible, and accountable to evidence-based standards across clinical and organizational settings.

### 4.5 Stakeholder communication, alliances, governmental affirmative actions, and conflict resolution: a roadmap for strategic partnership in decision making and implementation

Effective communication is essential for the success of initiatives at both the organizational and community levels. Engaging stakeholders, ensuring government support, and fostering collaborative partnerships can help overcome barriers, enhance relationships among stakeholders, and address conflicts through coordinated cooperation. This approach will facilitate the integration of Yoga into healthcare systems and communities. Two-way communication mechanisms must be established to foster transparency, trust, and collaboration among stakeholders. Symmetrical communication—where both parties contribute equally—may not always be feasible. Targeted communication to resolve conflict and address barriers would not be effective if common interests do not mediate the relationship among stakeholders.

Cost-effectiveness and cost-cutting measured goals for healthcare institutions could impose pressure on the systemically privileged entity that would help form a common interest and partnership, unfolding in strategic collaboration and integrating Yoga into healthcare systems. However, such moves would not result in equitable alliance and desired outcomes if a dedicated team of stakeholder representatives also participated in the newly designed Advisory Stakeholder Board and collaborated, analyzed, reflected, negotiated, and monitored implementation strategies being equitable, fair, and facilitative of targeted goals. The Advisory Stakeholder Board would deal with conflicts, resolutions, and data-driven decision-making mechanisms at different levels to avoid perspective-based decisions and facilitate evidence-based practice (EBP). All stakeholder representatives on the board must be voluntary, term-based, with no conflict of interest, bound by ethical practices, and knowledgeable/qualified people in integrative health approaches.

No matter how efficient and effective strategies are adopted, no linear methodology would help resolve conflict originating over funding, allocation, program design, and conflicting interests. To address this, rolling with resistance—a strategic conflict management approach—should be adopted. Key elements include regular stakeholder meetings, reliable digital information-sharing platforms, and phased public communication strategies to build community awareness and acceptance. With precise conflict resolution mechanisms at local, state, national, and international arenas, establishing clear communication strategies, a transparent decision-making roadmap, and simple conflict resolution steps can help in scoping review of progress and/or navigating setbacks to make the systems resilient and adaptive.

Furthermore, standardized guidelines and frameworks must be developed and adopted with periodic revisions to ensure uniformity in the application and strategic implementations of Yoga protocols, programs, adopted instructors' educational standards, partnership structures, and practice regulations. However, it would be challenging to integrate medical science protocols with Yoga practice unless a consistent evolutionary assimilation practice is monitored and tailored to emerging evidence and delivered through evidence-based practice models.

Adaptability and flexibility are crucial for successful program design and implementation. The stakeholder advisory board can adapt a program that can change its approach to better match the needs and expectations of stakeholders. On the other hand, a flexible program can modify its strategies in response to new evidence, input from stakeholders, and changes in external factors. These qualities are essential for programs that aim to achieve long-term success and positively impact the communities and individuals they serve. Programs can remain relevant and effective in an ever-changing world by being open to feedback and willing to make changes. However, the flexibility and adaptability of the system design must work under the framework of integrating Yoga with medical practice, facilitating collaboration between instructors and healthcare professionals in creating knowledge evidence, programs, training, integrated opportunities for future research and development, and their translation into evidence-based practice. Although a robust system and program design are essential, they will not flourish without government affirmative action and collaborative interests. These communication, alliance-building, and conflict-resolution strategies demonstrate that the integration of Yoga into healthcare systems ultimately depends on transparent governance structures, coordinated stakeholder engagement, and sustained governmental commitment, ensuring that implementation processes remain equitable, adaptive, and aligned with evidence-based practice across all levels of the system.

### 4.6 Implementation plan

To enhance the health and wellness of individuals, Yoga programs should be launched strategically in carefully selected healthcare institutions and community centers. Stakeholder input must be gathered during program development to ensure initiatives are tailored to the target populations' needs. Gathering input from stakeholders and integrating them into program development would prove beneficial. It would develop new partnerships to expand tailored interventions for the targeted populations. To ensure the program's success, it should be introduced gradually with adequate training and resources for healthcare personnel. The program's implementation should be closely supervised, and strategies should be adjusted based on stakeholder input and evaluation results. This approach ensures that the program is customized to stakeholders' needs and positively impacts the health of all participants.

### 4.7 Implementation strategies

The Implementation Science (IS) approach aims to revolutionize healthcare for patients by prioritizing patients' overall wellbeing and quality of life. The approach focuses on integrating non-pharmacological solutions with modern science to address physical and physiological ailments and mental health problems and enhance overall health-related quality of life (13). This can be achieved by promoting healthy lifestyles and self-management techniques, steering away from treatments that heavily rely on the disease model. The approach values the whole person, including their physical, psychological, emotional, and spiritual dimensions, utilizing the recovery and resilience models (13). The focus is to empower patients as decision-makers in their care following the EBP model, fostering active participation through coaching methods instead of the traditional hierarchical model. Other elements include:

- Advocating for inclusivity by involving families in health education and guidance.
- Developing learning opportunities for families and their wellbeing.
- Providing recommendations, protocols, and support services to healthcare workers working as an interdisciplinary team to ensure a comprehensive and compassionate approach to wellness promotion.

**Figure 1:**
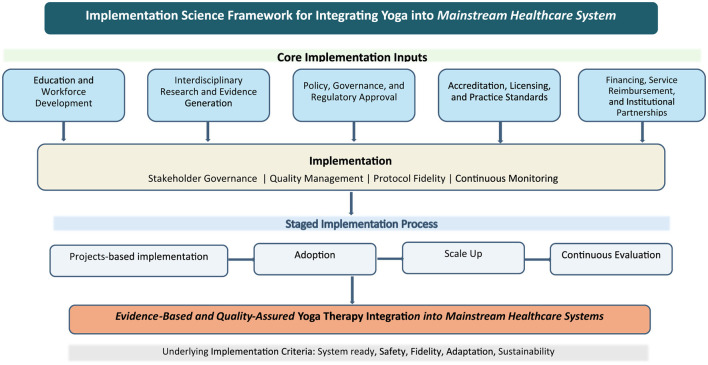
Implementation science framework for integrating Yoga into healthcare systems. The framework identifies core implementation inputs, including education and workforce development, interdisciplinary research and evidence generation, policy, governance, and regulatory approval, accreditation, licensing, and practice standards, and financing, reimbursement, and institutional partnerships. These inputs are coordinated through an implementation infrastructure comprising stakeholder governance, quality management, protocol fidelity, and continuous monitoring. The framework supports a staged implementation process from demonstration projects to adoption, scale-up, and continuous evaluation, leading to evidence-based and quality-assured yoga therapy integrated into healthcare systems.

Drawing on CCRYN–PGIMER stakeholder deliberations and the published implementation literature, a strategic framework was proposed for implementing Yoga as a recognized form of traditional medicine (TM) within healthcare systems (Figure 1).

To effectively integrate Yoga education, practice, research, and licensing, it is crucial to establish a standardized national approach that encompasses both physical, spiritual, and practical aspects of training following Yoga philosophy. To achieve this, there is a need to create a national body to oversee the integration (including standardization, approval, and regulation) of Yoga protocols for therapy, establish state boards to regulate its practice, and build collaborations with global entities. Additionally, involving businesses, regulatory bodies, and international partnerships to support Yoga initiatives is essential. Fundamentally, these suggestions strive to incorporate Yoga into healthcare, education, and research on a global scale—from local to international levels. The objective is to promote Yoga and guarantee its smooth organic integration into conventional healthcare, education, and research, encouraging a holistic approach to overall wellness.

The focus is on advancing research in traditional Yoga medicine while fostering interdisciplinary collaboration within an integrative health framework. This includes integration with fields such as medicine, molecular biology, microbiology, biotechnology as well as technology development in monitoring & communication devices, artificial intelligence (AI) initiatives, business and law at the national level, aiming for a cohesive approach that enhances the understanding of Yoga from the molecular to systems level. Additionally, committees are needed for policy review and public awareness campaigns through mass media and will take precedence over the implementation of Yoga in healthcare. Research grants-in-aid are also indispensable, and Yoga is incorporated into the competency-based educational, medical, and training curricula. These recommendations will extend to international platforms as well, with a focus on advocating for collaborative research and integrating Yoga into global health initiatives in conjunction with accreditation bodies at the international level, such as the International Association of Yoga Therapists (IAYT).

A comprehensive set of recommendations has been developed to envision the holistic integration of Yoga into healthcare, education, practice, and research. The recommendations have been drawn for grassroots state, national, and international level work from the international conference on Yoga and emerging barriers as an example of specific implementation science of Yoga in healthcare systems in India. At the grassroots level, the emphasis is on embedding Yoga in schools and colleges, professional degrees, and interdisciplinary professional and research programs such as MD and Ph.D. in Integrated Healthcare. Furthermore, community wellness centers, guided by trained workers like *Anganwadi* workers in India, are envisioned to expand their role, addressing diverse age groups and healthcare needs. State-level initiatives delve into integrating Yoga into medical institutions, with certificate courses in Integrative Medicine (as already initiated by the National Institute of Mental Health and Neurosciences, Bengaluru, India, in collaboration with Swami Vivekananda Yoga University, Bengaluru, India), parity in salaries for Yoga students, and mandatory courses for medical practitioners. Regulatory bodies are proposed, established, and promoted to standardize licensing exams and education, ensuring the quality of Yoga practice across states in addition to approval and regulation of Yoga protocols. This tier also envisions collaboration with international bodies for standardized protocols, highlighting the need for a cohesive approach. Additionally, integration is feasible without salary increases beyond parity with comparable health system roles, provided that training standards, supervision, and quality systems are established. Government bodies regulating the healthcare system must also promote job creation with specific requirements for such terminal degrees. Collectively, these multilevel recommendations highlight that the meaningful integration of Yoga into healthcare, education, research, and regulatory systems will require coordinated national and state structures, interdisciplinary collaboration, and sustained policy support, ensuring that Yoga is embedded as a credible, standardized, and institutionally anchored component of modern health systems.

### 4.8 Grassroots level recommendations

#### 4.8.1 Schools and colleges

- Introduce Yoga as part of the school curriculum.
- Incorporate basic breathing practices and postures in physical education.
- Promote Yoga as a sport/physical education to prevent opposition.

#### 4.8.2 Hospitals and primary health centers

- Start protocolized Yoga therapy sessions under trained Yoga therapists after applicable regulatory authorization and institutional quality oversight are in place.
- Expose medical professionals to Yoga practices and train them to advocate for Yoga practices during OPD visits.
- Promote awareness about evidence-based studies on Yoga.

#### 4.8.3 Community wellness centers

- Expand the role of *Anganwadi* workers to include teaching Yoga to various age groups.
- Establish community wellness centers for older adults and de-addiction programs.
- Provide basic Yoga training for teachers in schools.

### 4.9 State level recommendations

#### 4.9.1 Medical institutions

- Introduce Certificate Courses in Integrative Medicine for medical students and faculty.
- Include integrative medicine in MBBS and MD curricula. Start MD in Integrative Medicine and allow physicians to become “Integrative physicians” by completing a bridge course in Integrative Medicine.
- Allow Integrative Medicine professionals to practice in outpatient departments (OPDs).

#### 4.9.2 Health departments

- Implement comparative monetary support for Yoga students compared to MBBS students.
- Make 1-week assessment-based introductory Yoga courses mandatory for medical students.
- Empower the Ministry of Ayush to grant funds for Yoga research beyond PGI.

#### 4.9.3 Regulatory bodies

- Standardize Yoga licensing exams and education through a national accreditation body.
- Create state boards to regulate Yoga therapists' ethical and professional behaviors, increase accountability, and reinforce the need for continuing education requirements.
- Establish or work with existing regulatory bodies, such as the Drug Controller General of India (DCGI), to oversee the regulation and approval of Yoga protocols for therapy.
- A central regulatory authority responsible for reviewing, approving, and periodically updating Yoga therapeutic protocols must be incorporated into the governance architecture. Its function would parallel that of the DCGI by reviewing evidence, verifying methodological rigor, and authorizing approved protocols for clinical use. The presence of such a regulatory body is essential for transparency, quality assurance, and legitimacy within conventional healthcare environments.
- Regulatory approval of Yoga therapeutic protocols should be mandated prior to clinical integration. Alignment with the DCGI or an equivalent regulatory mechanism is necessary to ensure that all approved protocols meet defined standards of safety, evidence, and therapeutic fidelity.
- Collaborate with international accreditation and regulatory bodies for standardized protocols and approvals for medical use.

### 4.10 National level recommendations

#### 4.10.1 Research and education

- Enhance research aptitude in AYUSH professionals through workshops and CMEs.
- Open AYUSH faculty posts in all INIs with structured courses on Integrative Medicine.
- Formulate short-term Integrative Medicine certificate courses at central and state universities.

#### 4.10.2 Health policy and planning

- Set up a review committee to track progress and modify health policies.
- Include Yoga courses in the National Medical Commission's competency-based curricula.
- Restart Research Grants-in-aid for Yoga research with stringent evaluation methods.

#### 4.10.3 Public awareness

- Use mass media to highlight the role of Yoga in various diseases.
- Incorporate Yoga into school and higher education curricula.
- Initiate Yoga awareness programs for patients and doctors.

### 4.11 International level recommendations

#### 4.11.1 Research collaboration

- Collaborate with global platforms like UN/WHO for Yoga awareness.
- Fund overseas Yoga research programs and promote international collaboration.
- Collaborate with international accreditation and regulatory bodies such as MHRA (UK) and FDA (US) for standardized protocols and their approval for medical use.

#### 4.11.2 Global health initiatives

- Include Yoga in global health initiatives for mental health and wellbeing.
- Encourage international businesses to contribute to Yoga research.
- Collaborate with lifestyle professional bodies for mainstream clinical practice.

### 4.12 Yoga education, practice, research, and licensing

#### 4.12.1 Education and practice

- Standardize Yoga knowledge, practice, and protocols at the national level.
- Incorporate Yoga into modern medicine curricula at schools and MBBS levels.
- Emphasize the spiritual dimension of Yoga in training and practice.

#### 4.12.2 Research and licensing

- Establish a national body to oversee the integration of Yoga into modern medicine.
- Create state boards to regulate Yoga practice and licensing.
- Standardize licensing exams and create a national accreditation body.
- Approval of Yoga therapeutic protocols must be grounded in evidence demonstrating safety, effectiveness, and reproducibility. Standardization, while necessary for consistency of delivery and training, does not constitute approval. Regulatory authorization should rely on empirical validation, including clinical trials and translational evidence, ensuring that protocols meet the evidentiary threshold required for integration into medical practice.
- Training modules for Yoga therapists and clinicians should reference regulator-approved, evidence-supported protocols. Standardized curricula must therefore be continually aligned with evolving evidence and regulatory decisions rather than relying solely on traditional or pedagogical conventions.

#### 4.12.3 Collaboration and funding

- Collaborate with other disciplines and regulatory bodies for integrative health.
- Create a database of public and private funders for Yoga research.
- Encourage businesses to contribute to Yoga initiatives as part of corporate social responsibility.

### 4.13 Key components in the implementation science of Yoga

The implementation science of Yoga involves systematically studying and applying strategies to integrate evidence-based Yoga practices into diverse settings, ensuring effectiveness, sustainability, and scalability. Here's an overview of key components in the implementation science of Yoga:

#### 4.13.1 Assessment and planning

- Needs Assessment: Identify the target population and their specific health needs that can be addressed through Yoga interventions.
- Contextual Analysis: Understand the organizational and cultural context to tailor Yoga programs accordingly.
- Resource Assessment: Evaluate the availability of trained Yoga instructors, facilities, and community support.

#### 4.13.2 Intervention design

- Evidence-Based Practices: Utilize scientific evidence to design Yoga interventions with proven health benefits.
- Tailoring: Customize interventions to match the characteristics and preferences of the target population.
- Adaptability: Design programs adapted to different settings, populations, and cultural contexts.

#### 4.13.3 Training and capacity building

- Instructor Training: Ensure that Yoga instructors are well-trained, certified, and capable of delivering evidence-based interventions.
- Capacity Building: Train healthcare professionals, educators, and community leaders to integrate Yoga into their practices.

#### 4.13.4 Implementation strategies

- Behavioral Change Models: Apply behavioral change theories to understand and influence individuals' and organizations' readiness to adopt Yoga practices.
- Implementation Frameworks: Use established frameworks such as the “Consolidated Framework for Implementation Research (CFIR)” to guide the implementation process (8).

#### 4.13.5 Evaluation and monitoring

- Outcome Measures: Define clear and measurable outcomes to assess the impact of Yoga interventions on physical, mental, and emotional wellbeing.
- Process Evaluation: Continuously monitor the fidelity and quality of Yoga program delivery to ensure adherence to evidence-based practices.
- Use technological advancements in monitoring, measurement methods, diagnostics, and AI for continuous evaluation, compliance and monitoring effectiveness.

#### 4.13.6 Stakeholder engagement

- Community Involvement: Engage communities, patients, and relevant stakeholders in the planning and implementation.
- Cultural Sensitivity: Respect and incorporate cultural nuances to enhance the acceptability of Yoga interventions.

#### 4.13.7 Policy advocacy

- Integration into Policies: Advocate for including Yoga in healthcare and educational policies at various levels.
- Research Translation: Translate scientific evidence into policy recommendations by bridging the gap between research findings and practical application.

#### 4.13.8 Technology integration

- Digital Platforms: Explore the use of technology, such as mobile apps and online platforms, new technological devices, including those using AI, to facilitate remote access to Yoga programs and feedback from users, patients, and practitioners.
- Data Analytics: Utilize data analytics to track participant engagement, assess program effectiveness, and make data-driven improvements.

#### 4.13.9 Long-term sustainability

- Community Empowerment: Foster a sense of community ownership and empowerment to sustain Yoga programs beyond initial implementation.
- Organizational Integration: Integrate Yoga into existing healthcare, educational, and community structures for long-term institutionalization.

#### 4.13.10 Dissemination and knowledge translation

- Educational Campaigns: Conduct awareness campaigns to disseminate information about the benefits of Yoga.
- Knowledge Exchange: Facilitate the exchange of knowledge and best practices among stakeholders, researchers, and practitioners.

The implementation of the science of Yoga seeks to bridge the gap between scientific research, education, and training to foster their seamless integration into patients' care practices through diverse healthcare settings to improve the overall health and wellbeing of the community. However, a feedback and evaluation system will be required to streamline the process and monitor outcomes. These domains underscore that the effective implementation of Yoga within healthcare systems requires coordinated development across education, practice, research, licensing, policy advocacy, and long-term sustainability structures, ensuring that evidence-based Yoga interventions are systematically integrated, contextually adapted, and institutionally supported as a coherent component of modern healthcare.

### 4.14 Monitoring and evaluation of Yoga implementation

To ensure the efficient implementation of a program and the active participation of its stakeholders, continuous monitoring and evaluation of the program's progress, outcomes, and stakeholder satisfaction are essential for the successful integration of Yoga and optimal functioning of the integrated healthcare systems. It is necessary to employ quantitative and qualitative evaluation methods to assess program outcomes and identify areas for improvement. Additionally, periodic review meetings with stakeholders can provide valuable insights and opportunities to make necessary adjustments. Prioritizing these steps can help program managers maximize the impact of their initiatives and ensure that they deliver the intended benefits to all stakeholders involved. However, it may not be effective if it is not integrated well at the policy level with different bodies, such as accreditation and licensing bodies. Such agencies are responsible for reinforcing accountability and controlling malpractices. Thus, sustained monitoring and evaluation will be effective only when aligned with broader regulatory and policy structures, ensuring accountability, standardization, and long-term institutional integration of Yoga-based programs within the healthcare system.

Furthermore, new technology developments such as in sensing and monitoring devices, and multiomics technologies that encompass genomics, transcriptomics, proteomics, metabolomics, microbiomics, spectromics, and AI, can offer a powerful framework for evaluating individual responses to Yoga-based interventions across various disease conditions (14). Given that Yoga modulates physiological systems also targeted by pharmacological agents, it is plausible that inter-individual variability in therapeutic outcomes may mirror patterns observed in pharmacogenomics (15). In clinical research, multiomics can be helpful in stratifying participants based on molecular signatures, enabling personalized approaches to Yoga therapy. However, we suggest incorporating traditional yogic concepts like *Triguna* (*Sattva, Rajas, Tamas*) and *Tridosha* (*Vata, Pitta, Kapha*), which capture psychophysiological constitutions influencing intervention responses, to achieve more nuanced and holistic stratification—particularly in Yoga's experiential framework. These can be operationalized along with multiomics data. For example, baseline evaluations of *prakriti* (constitutional type) using validated questionnaires may correlate with omic profiles (such as metabolomic markers of *doshic* imbalance or epigenetic signatures associated with guna dominance), as shown in Ayurgenomics research conducted by Mukerji et al. (29) who investigated the genomic foundations of Ayurvedic prakriti for precision medicine. This cooperative strategy improves efficacy and relevance without replacing multiomics validation by bridging traditional knowledge with contemporary science.

These sensors, such as those captured by sensor-embedded Yoga mats, could enable real-time feedback and adaptive practice. The integration of molecular evidence with digital health technologies presents significant potential for advancing the field of precision Yoga. This interdisciplinary approach not only aims to enhance participant engagement but also facilitates improved self-regulation and therapeutic optimization. By leveraging molecular data in conjunction with digital tools, researchers and practitioners can tailor Yoga interventions to meet the specific needs of individuals, thereby fostering a more effective and personalized health experience.

### 4.15 Implementation in healthcare settings

Implementing Yoga in all healthcare hospitals, educational institutions, research programs, and community settings has significant potential to transform healthcare delivery while improving patient outcomes. Our analysis suggests that integrating Yoga into healthcare can improve patient experience and potentially reduce system costs through prevention and adjunctive benefits, provided that implementation draws on stakeholder governance, economic analysis, and quality management (3, 16). However, given heterogeneity in protocols, populations, and outcomes, effects should be interpreted as condition-specific and context-dependent, rather than universally generalizable. Findings are consistent with the implementation science aim of closing the know-do gap by aligning evidence-based practice with real-world adoption through governance, financing, and quality systems.

#### 4.15.1 Multisector collaboration: the foundation for success

The successful implementation of Yoga in healthcare systems requires a multisector collaboration among government bodies, medical and Yoga science professionals, healthcare institutions, and community stakeholders. Government health departments play a vital role in providing essential policy support, funding, and regulatory frameworks to integrate Yoga into healthcare systems while ensuring transparency in decision-making processes (12, 17). Medical and Yoga science professionals contribute expertise and evidence-based protocols that are essential for credibility within healthcare settings. Healthcare institutions and community members provide implementation settings and critical feedback mechanisms that ensure interventions remain relevant and effective (17, 18). This collaborative network, operating at local, state, national, and international levels through advisory boards and quality control mechanisms, can facilitate the seamless integration of Yoga into modern healthcare (19).

#### 4.15.2 Evidence-based practice: the core of integration

For Yoga to be integrated into the mainstream healthcare system, yoga professionals will need to follow the framework of evidence-based practice (EBP) to improve clinical outcomes in patients (3). To engage in evidence-based practice, the yoga service providers will have to identify the best evidence-informed therapeutic model suited to the patients' current conditions, incorporate their preferences and needs, and assess the treatment viability within available resources and clinical expertise before making clinical decisions about the interventions. The literature indicates that standardized protocols aligned with quality assessment and assurance through regulatory bodies can significantly increase the adoption rate of therapeutic yoga practices in mainstream health care systems (20, 21). The democratization of evidence-based Yoga protocols through reliable, accessible sources will accelerate acceptance and implementation across diverse healthcare settings. Research has shown that without this evidence base, integration efforts will face significant resistance from medical establishments, particularly in resource-limited settings where competing priorities may exist (17). However, to effectively integrate Yoga in mainstream healthcare systems, it would be ideal to adopt the collaborative model of social workers' services as used within the extensive healthcare network in the U.S. This includes training social workers to understand the benefits of Yoga as an alternative therapy, allowing them to assess patient needs and make appropriate referrals. Establishing multidisciplinary teams with social workers, Yoga therapists, and healthcare providers can facilitate holistic patient care. Also, social workers trained in medical and psychiatric social work and community organizing can play a key role in community outreach by organizing accessible Yoga programs and ensuring cultural competence in practice, ultimately enhancing patient engagement and health outcomes.

#### 4.15.3 Communication: building trust and engagement

Transparent communication among all stakeholders represents a critical success factor for implementation. Effective Yoga integration requires culturally sensitive two-way communication between patients and practitioners, clear information pathways between healthcare institutions and Yoga providers, and feedback loops at multiple implementation stages to address challenges promptly (11). Knowledge translation strategies to make research findings accessible to non-specialists are also essential for wider adoption. These communication channels build trust, enhance belief in new health approaches, and foster effective utilization of Yoga services at the community level (22). Moreover, Yoga practice frameworks naturally facilitate social capital development, which research has shown is essential for community health and resilience (8). The practice framework of Yoga would also facilitate communication and social capital, which are essential for a community to thrive. However, communication must have a feedback loop at different stages to ensure it is reciprocal and progressive in addressing the discomfort and distress of patients quickly in the newly established Yoga-medicine partnership (23). Service models similar to social workers in the U.S. can help enhance patient communication, improve outcomes, and facilitate discharge planning for individuals facing various biopsychosocial challenges, while also alleviating mental and physical barriers to accessing Yoga services.

#### 4.15.4 Quality management

A comprehensive quality management approach is essential for sustainable implementation. Yoga instructors and practitioners should follow standardized protocols and undergo regular evaluations to maintain quality standards following evidence-based practice and clinical decision-making (3). Standardized protocols and evaluations are important for quality control. The purpose of Yoga therapy is to provide holistic health, intuitive integration of *asanas*, breathwork, and meditation, which requires personal mastery and attention to individual indicators such as autonomic shifts or emotional states that rigorous checklists miss. Instructors require more for therapeutic success in illnesses such as cardiovascular disease, including guru-guided mentorship, clinical apprenticeships, self-reflective practice, and biological training (e.g., HRV or inflammatory markers). This multimodal method provides safe and transformative healing that goes beyond mechanical compliance.

Regular evaluation of Yoga programs against established benchmarks, systematic tracking of outcomes with appropriate blinding to minimize bias, and data systems supporting auditability and verifiability are crucial elements of quality assurance (16). Healthcare institutions must allocate adequate resources and establish appropriate infrastructure to support Yoga programs while maintaining rigorous quality standards. Educational institutions have a critical role in developing standardized curricula and archiving educational materials for widespread use, as demonstrated in successful implementation models (8, 24). Studies have shown that quality management is particularly important in maintaining fidelity to interventions while allowing for necessary adaptations to local contexts (7, 25).

#### 4.15.5 Economic factors: creating sustainable financing models

The economic dimension of Yoga implementation cannot be overlooked. Insurance companies can play a decisive role by including evidence-based Yoga interventions in coverage plans and creating reimbursement models for Yoga therapy services (17). Support for data collection on cost-effectiveness and incentivization of preventative approaches through premium structures has shown promise in pilot programs (26). Research institutions must continue generating evidence on the cost-effectiveness of Yoga interventions compared to conventional treatments for various conditions. Studies comparing cost-benefit analysis, cost-effectiveness analysis, and cost-utility analysis provide essential information for decision-makers considering integrating Yoga into healthcare systems (5, 6, 14). This economic case strengthens the rationale for implementation across different healthcare settings, particularly in resource-constrained environments where maximizing healthcare value is imperative (9).

#### 4.15.6 Looking forward: a roadmap for implementation

Moving forward, we propose a phased approach to implementation that begins with establishing demonstration projects in select healthcare settings with comprehensive evaluation, followed by scaling successful models with appropriate adaptations for different contexts. The subsequent integration phase would incorporate Yoga permanently into healthcare structures with sustainable funding, leading to an innovation phase where approaches are continuously refined based on emerging evidence and feedback (10, 17). Successful integration models, such as the one at NIMHANS, where conventional medical practitioners and Yoga therapists collaborate in treatment planning and delivery, demonstrate the feasibility of this approach when implemented with attention to institutional context and collaborative practice (19). Throughout this process, stakeholder engagement remains central, ensuring that implementation responds to the needs of patients, providers, and healthcare systems while maintaining fidelity to Yoga's traditional principles. International collaboration through established bodies and the World Health Organization can facilitate knowledge sharing and standardization of approaches across borders (12).

By addressing these interconnected dimensions of implementation science, Yoga can move from the periphery to become an integral component of modern healthcare systems, offering patients complementary approaches to health and wellness while potentially reducing the overall burden on healthcare resources (30). The integration framework proposed in this paper provides a comprehensive roadmap for policymakers, healthcare administrators, and practitioners seeking to implement Yoga in diverse healthcare settings. Future research should focus on evaluating the outcomes of this implementation framework across different cultural and healthcare contexts, refining the approach based on evidence and stakeholder feedback, and developing more sophisticated models for integrating traditional medicine practices into contemporary healthcare systems (17, 18).

#### 4.15.7 Implications and recommendations

Throughout this manuscript, community and school Yoga initiatives refer to wellness-oriented programming, whereas Yoga delivered as a therapeutic clinical protocol requires prior regulatory authorization, protocol-specific training, and quality oversight before routine clinical integration. Recommendations are classified as evidence-informed when supported by published sources and as consensus-based when primarily informed by stakeholder deliberations or expert judgment in areas where published implementation evidence is sparse. We have differentiated between evidence-informed items backed by moderate to robust evidence or established implementation principles, exploratory or consensus-based items, and items based on emerging evidence or expert opinion.

##### 4.15.7.1 Evidence-informed recommendations

- Establish institution-level Advisory Stakeholder Boards with clear terms of reference, including conflict-of-interest policies, defined term limits, and explicit remit over protocol standardization and outcome review (16, 17). For example, a tertiary hospital board co-chaired by a clinician and a Yoga therapy lead, meeting quarterly to review fidelity indicators and authorize protocol revisions.
- Launch phased demonstration projects in priority service lines (e.g., cardiac rehabilitation, oncology supportive care, chronic pain clinics) with predefined stop-go-adapt criteria to guide scale-up decisions (20, 21, 27). For example, a 6-month pilot with predefined thresholds for enrollment, adherence, and clinically meaningful outcomes to inform progression decisions.
- Between demonstration projects and large-scale expansion, an intermediate adoption phase is required. Adoption should occur only after regulatory approval has been granted, feasibility has been verified, and evidence from demonstration projects has been evaluated. This phase includes institution-level endorsement, workforce orientation, integration into clinical workflows, and verification of infrastructure readiness. Only after successful adoption should scale-up proceed, ensuring alignment with implementation science constructs of readiness, fidelity, and sustainability.
- Use demonstration projects to raise funding and launch wider evidence-generating clinical trials for establishing effectiveness, regulatory submissions, and approvals.
- Implement quality management systems for fidelity monitoring, outcome tracking, and auditability, with explicit linkage to institutional risk management and learning cycles (3, 16, 18). Example: standardized session checklists, adverse-event logs, and quarterly audits conducted by designated quality teams.
- Align financing models with prevention and adjunctive-care incentives, informed by evidence on feasibility, cost-effectiveness signals for specific indications, and health-system integration of Yoga-based interventions (2, 3, 21). For example, payer-provider arrangements that support reimbursement for protocolized, evidence-based Yoga adjuncts for conditions such as chronic low back pain or anxiety disorders.
- Conduct structured assessments of organizational readiness prior to implementation, including leadership engagement, workforce capacity, workflow integration, and infrastructure adequacy, to inform sequencing and resource allocation (25, 28). For example, readiness assessments are used to determine whether services proceed directly to piloting or require preparatory capacity-building.
- Ensure standardized workforce training, competency verification, and supervision for Yoga therapists and collaborating clinicians, aligned with effective and evidence based protocols and institutional safety standards (3, 8, 16). For example, protocol-specific training followed by supervised practice and periodic competency review.
- Integrate Yoga interventions into institutional data systems, including electronic health records, registries, and dashboards, using standardized documentation to support monitoring, auditability, and reimbursement processes (16, 18). For example, structured EHR templates capture session content, adherence, adverse events, and outcomes.
- Establish structured adaptation processes that allow context-sensitive modification of protocols while documenting rationale, scope, and implications for fidelity and outcomes (16, 25). For example, formally reviewed protocol amendments approved by the advisory board following fidelity and outcome review.
- Predefine and monitor implementation outcomes alongside clinical outcomes, including adoption, feasibility, acceptability, fidelity, penetration, and sustainability, to guide iterative refinement and scale-up decisions (25, 28). For example, routine reporting of adoption rates and fidelity metrics during pilot and early scale-up phases.

##### 4.15.7.2 Exploratory consensus-based recommendations

- Create state-level boards to regulate Yoga practice and licensing, aligned with medical accreditation bodies and relevant international associations, and develop bridging curricula for integrative clinicians to facilitate interdisciplinary practice. *Potential constraint:* statutory alignment across jurisdictions and workforce supply.
- Incorporate novel technological advancements, multiomics approaches, digital biomarkers, and AI to personalize Yoga-based interventions and provide real-time adherence and response feedback. *Potential constraint:* costs, infrastructure requirements, and the current maturity of the evidence base.
- Scale community wellness centers and school-based programs using tiered training and supervision models for frontline workers to extend reach while maintaining basic quality safeguards. *Potential constraint:* training capacity, supervision models, and long-term sustainability.
- Clarify legal, liability, and scope-of-practice frameworks for integrative Yoga delivery across clinical, community, and educational settings. *Potential constraint:* variability in medico-legal statutes, professional indemnity arrangements, and institutional risk tolerance.
- Establish national and international coordination mechanisms to harmonize Yoga education standards, Yoga protocol accreditation and approval processes, and clinical practice frameworks across jurisdictions. *Potential constraint:* inter-agency coordination challenges and cross-national regulatory variation.
- Develop coordinated public communication and mass-media strategies to support informed acceptance, appropriate utilization, and trust in the integration of Yoga within healthcare systems. *Potential constraint:* message consistency, credibility, and susceptibility to misinformation.

Workforce training, legal and insurance alignment, data governance, and cultural adaptation are likely to constitute rate-limiting factors; accordingly, implementation plans should incorporate dedicated provisions for staged capacity building, legal and regulatory review, and sustained community engagement.

## 5 Limitations

This structured narrative review has limitations. Table 1 classifications were coded descriptively using title cues and PubMed record fields, and the synthesis prioritized implementation relevance rather than exhaustive clinical effectiveness coverage. While Supplementary Table S1 preserves the auditability of the PubMed search yield, targeted non-PubMed sources (policy and institutional materials) were used for contextual framing and are not intended to represent a comprehensive gray literature systematic search. These limitations should be considered when interpreting the scope and generalizability of implementation gaps identified. Stakeholder deliberations were used only as contextual input; no unpublished proceedings were analyzed or treated as evidence.

## 6 Conclusion

This review proposes a pragmatic implementation framework that integrates stakeholder governance, economic evaluation, and quality-managed piloting to support the systematic integration of Yoga into healthcare systems. By moving beyond questions of clinical efficacy, the framework advances the literature toward operationalizing Yoga within real-world healthcare contexts and provides a structured, adaptable template for institutional adoption. The synthesis demonstrates that successful integration depends on coordinated action across governance, financing, quality management, and phased implementation strategies grounded in evidence-based practice and core constructs of implementation science, including readiness, fidelity, adaptation, and sustainability.

The framework highlights that while empirical evidence can inform protocol design, piloting, and outcome monitoring, broader system-level enablers such as regulatory alignment, workforce development, legal and liability clarity, and public communication often rely on expert consensus and policy coordination. Together, evidence-informed and consensus-based components are necessary to support scalable, accountable, and context-sensitive integration of Yoga across institutional, state, and national health systems.

Although persistent barriers remain, including policy heterogeneity, lack of implementation structures, accreditation gaps, workforce constraints, and reimbursement variability, the framework offers a coherent pathway for addressing these challenges through staged implementation, governance oversight, and continuous learning cycles. The recommendations presented here are intended to guide healthcare institutions, policymakers, and implementers in designing, piloting, and scaling Yoga-based interventions in a manner that is clinically credible, economically defensible, and systemically sustainable.

Future research should prioritize the development of condition-specific implementation pathways supported by rigorous evaluation of implementation outcomes, cost-effectiveness, and long-term sustainability across diverse healthcare settings. Multicenter studies examining fidelity, safety, effectiveness, and real-world adaptability of standardized Yoga protocols are needed, alongside research on regulatory models, reimbursement mechanisms, and interprofessional training approaches. Advancing Yoga from a supportive adjunct to an institutionally embedded component of evidence-based healthcare will require continued alignment between empirical evidence, implementation science, and coordinated policy action.
