# Supplementary material for: Implementation science of Yoga: an integrated approach to health and wellness
Source: Front Public Health. 2026 Jul 23;14:1757700. doi: 10.3389/fpubh.2026.1757700 (PMC13446387; doi:10.3389/fpubh.2026.1757700)
Supplement: Supplementary file 1 [file Table_1.docx]

**Supplementary Table S1. Complete PubMed Search Result (n = 143).**

Complete PubMed search results (n = 143) retrieved using yoga and implementation-relevant search terms (implementation, integration, feasibility, acceptability, fidelity, adoption, policy, governance, reimbursement, and service delivery), limited to human studies published in English. Provided to preserve auditability of the search yield. The implementation-explicit subset (n = 59) is presented in Table 1.

| PMID | First Author | Year | Title | Journal | Setting | DOI | Citation |
| --- | --- | --- | --- | --- | --- | --- | --- |
| 38035307 | Li Y | 2023 | Exercise intervention for patients with chronic low back pain: a systematic review and network meta-analysis | Front Public Health | Other/Mixed | 10.3389/fpubh.2023.1155225 | Front Public Health. 2023 Nov 17;11:1155225. doi: 10.3389/fpubh.2023.1155225. eCollection 2023. |
| 20105062 | Ross A | 2010 | The health benefits of yoga and exercise: a review of comparison studies | J Altern Complement Med | Other/Mixed | 10.1089/acm.2009.0044 | J Altern Complement Med. 2010 Jan;16(1):3-12. doi: 10.1089/acm.2009.0044. |
| 32463708 | Rousseau D | 2020 | Exploring the Benefit of Yoga Programs in Carceral Settings | Int J Yoga Therap | Justice/Carceral | 10.17761/2020-D-19-00039 | Int J Yoga Therap. 2020 Jan 1;30(1):111-115. doi: 10.17761/2020-D-19-00039. |
| 39485352 | Tankha H | 2024 | Effectiveness of Virtual Yoga for Chronic Low Back Pain: A Randomized Clinical Trial | JAMA Netw Open | Healthcare | 10.1001/jamanetworkopen.2024.42339 | JAMA Netw Open. 2024 Nov 4;7(11):e2442339. doi: 10.1001/jamanetworkopen.2024.42339. |
| 20188996 | Hayes M | 2010 | Prescribing yoga | Prim Care | Other/Mixed | 10.1016/j.pop.2009.09.009 | Prim Care. 2010 Mar;37(1):31-47. doi: 10.1016/j.pop.2009.09.009. |
| 28957795 | Datey P | 2018 | Combined Ayurveda and Yoga Practices for Newly Diagnosed Type 2 Diabetes Mellitus: A Controlled Trial | Complement Med Res | Other/Mixed | 10.1159/000464441 | Complement Med Res. 2018;25(1):16-23. doi: 10.1159/000464441. Epub 2017 Sep 29. |
| 36445191 | Kligler B | 2023 | What We Have Learned About the Implementation of Whole Health in the Veterans Administration | J Integr Complement Med | Healthcare | 10.1089/jicm.2022.0753 | J Integr Complement Med. 2023 Dec;29(12):774-780. doi: 10.1089/jicm.2022.0753. Epub 2022 Nov 23. |
| 31519272 | Nauphal M | 2019 | Yoga for the treatment of depression: Five questions to move the evidence base forward | Complement Ther Med | Other/Mixed | 10.1016/j.ctim.2019.08.012 | Complement Ther Med. 2019 Oct;46:153-157. doi: 10.1016/j.ctim.2019.08.012. Epub 2019 Aug 16. |
| 34198737 | Sun Y | 2021 | Yoga and Mindfulness Interventions for Preschool-Aged Children in Educational Settings: A Systematic Review | Int J Environ Res Public Health | Education | 10.3390/ijerph18116091 | Int J Environ Res Public Health. 2021 Jun 5;18(11):6091. doi: 10.3390/ijerph18116091. |
| 26535474 | Butzer B | 2015 | School-based Yoga Programs in the United States: A Survey | Adv Mind Body Med | Education |  | Adv Mind Body Med. 2015 Fall;29(4):18-26. |
| 35105638 | Ward L | 2022 | Development of the CLARIFY (CheckList stAndardising the Reporting of Interventions for Yoga) guidelines: a Delphi study | BMJ Open | Other/Mixed | 10.1136/bmjopen-2021-054585 | BMJ Open. 2022 Jan 31;12(1):e054585. doi: 10.1136/bmjopen-2021-054585. |
| 37752520 | Price J | 2023 | Women's experiences with yoga after a cancer diagnosis: a qualitative meta-synthesis-part I | Syst Rev | Other/Mixed | 10.1186/s13643-023-02350-x | Syst Rev. 2023 Sep 26;12(1):176. doi: 10.1186/s13643-023-02350-x. |
| 34076530 | Zhang Y | 2021 | Increasing Trend of Yoga Practice Among U.S. Adults From 2002 to 2017 | J Altern Complement Med | Other/Mixed | 10.1089/acm.2020.0506 | J Altern Complement Med. 2021 Sep;27(9):778-785. doi: 10.1089/acm.2020.0506. Epub 2021 Jun 1. |
| 38155602 | Ishaq J | 2023 | A Pilot Study of Yoga with Incarcerated Youth Using the Prison Yoga Project Approach | Int J Yoga Therap | Justice/Carceral | 10.17761/2023-D-23-00012 | Int J Yoga Therap. 2023 Dec 1;33(2023):Article 16. doi: 10.17761/2023-D-23-00012. |
| 32234747 | Alvarez E | 2020 | Evaluating a group-based Yoga of Stress Resilience programme: a pragmatic before-and-after interventional study protocol | BMJ Open | Healthcare | 10.1136/bmjopen-2019-035862 | BMJ Open. 2020 Mar 31;10(3):e035862. doi: 10.1136/bmjopen-2019-035862. |
| 34210638 | Kaur S | 2022 | Evaluation of an integrated yoga program in patients with inflammatory bowel disease: A pilot study | Explore (NY) | Healthcare | 10.1016/j.explore.2021.04.006 | Explore (NY). 2022 May-Jun;18(3):335-341. doi: 10.1016/j.explore.2021.04.006. Epub 2021 May 4. |
| 34301513 | Kanchibhotla D | 2022 | Impact of Prajñā yoga on cognition in adolescents with congenital and adventitious visual impairment | Explore (NY) | Other/Mixed | 10.1016/j.explore.2021.05.010 | Explore (NY). 2022 May-Jun;18(3):313-318. doi: 10.1016/j.explore.2021.05.010. Epub 2021 Jul 5. |
| 41356177 | Zunin ID | 2025 | Effects of Tummo Meditation and Niguma Yoga on Brain Activity | Hawaii J Health Soc Welf | Other/Mixed | 10.62547/DNMT3002 | Hawaii J Health Soc Welf. 2025 Sep;84(9):195-199. doi: 10.62547/DNMT3002. |
| 38151281 | Kashyap K | 2023 | Yoga therapy on elderly patients with fear of fall: an open-label randomised controlled trial (YOFEAR trial) | BMJ Open | Other/Mixed | 10.1136/bmjopen-2022-070540 | BMJ Open. 2023 Dec 27;13(12):e070540. doi: 10.1136/bmjopen-2022-070540. |
| 40454738 | Ghosh M | 2025 | Scope and Role of Integrating Yoga in Homeopathic Medical Education: An Explorative Review | Altern Ther Health Med | Education |  | Altern Ther Health Med. 2025 Sep;31(5):234-241. |
| 38155603 | Telles S | 2023 | Patients' Choices for Yoga Therapy: An Exploratory Cross-Sectional, Convenience-Sampling Survey from India | Int J Yoga Therap | Other/Mixed | 10.17761/2023-D-22-00087 | Int J Yoga Therap. 2023 Dec 1;33(2023):Article 15. doi: 10.17761/2023-D-22-00087. |
| 36731199 | Sharpe E | 2023 | A closer look at yoga nidra- early randomized sleep lab investigations | J Psychosom Res | Other/Mixed | 10.1016/j.jpsychores.2023.111169 | J Psychosom Res. 2023 Mar;166:111169. doi: 10.1016/j.jpsychores.2023.111169. Epub 2023 Jan 29. |
| 33528905 | Jakicic JM | 2021 | Feasibility of Integration of Yoga in a Behavioral Weight-Loss Intervention: A Randomized Trial | Obesity (Silver Spring) | Other/Mixed | 10.1002/oby.23089 | Obesity (Silver Spring). 2021 Mar;29(3):512-520. doi: 10.1002/oby.23089. Epub 2021 Feb 2. |
| 38751579 | Santos S | 2024 | The effect of Ashtanga-Vinyasa Yoga method on air force pilots' operational performance | Front Public Health | Other/Mixed | 10.3389/fpubh.2024.1334880 | Front Public Health. 2024 May 1;12:1334880. doi: 10.3389/fpubh.2024.1334880. eCollection 2024. |
| 32300055 | Taylor J | 2020 | Personalised yoga for burnout and traumatic stress in junior doctors | Postgrad Med J | Other/Mixed | 10.1136/postgradmedj-2019-137413 | Postgrad Med J. 2020 Jun;96(1136):349-357. doi: 10.1136/postgradmedj-2019-137413. Epub 2020 Apr 16. |
| 31436394 | Wimmer L | 2019 | Improving emotion regulation and mood in teacher trainees: Effectiveness of two mindfulness trainings | Brain Behav | Education | 10.1002/brb3.1390 | Brain Behav. 2019 Sep;9(9):e01390. doi: 10.1002/brb3.1390. Epub 2019 Aug 22. |
| 35377949 | Ahmadi E | 2022 | Assessing the Acceptability of Yoga Among Patients with and without Chronic Pain Enrolled in a Licensed Opioid Treatment Program | Int J Yoga Therap | Healthcare | 10.17761/2022-D-21-00055 | Int J Yoga Therap. 2022 Jan 1;32(2022):Article 3. doi: 10.17761/2022-D-21-00055. |
| 36795229 | Addington EL | 2023 | Qualitative similarities and distinctions between participants' experiences with a yoga intervention and an attention control | Support Care Cancer | Other/Mixed | 10.1007/s00520-023-07639-0 | Support Care Cancer. 2023 Feb 16;31(3):172. doi: 10.1007/s00520-023-07639-0. |
| 37169721 | Cheruka CA | 2023 | Oxygen Consumption and Heart Rate Responses in Different Vinyasa Yoga Sequences | Int J Yoga Therap | Other/Mixed | 10.17761/2023-D-22-00058 | Int J Yoga Therap. 2023 Jan 1;33(2023):Article 2. doi: 10.17761/2023-D-22-00058. |
| 39313765 | Loy M | 2024 | Implementation of Virtual Yoga Shared Medical Appointment (VYSMA) Pilot at an Academic Medical Center Within a Mixed-Diagnosis Oncology Population | Int J Yoga Therap | Healthcare | 10.17761/2024-D-24-00004 | Int J Yoga Therap. 2024 Sep 1;34(2024):Article 12. doi: 10.17761/2024-D-24-00004. |
| 39066651 | Saint K | 2024 | Assessing Fidelity in Yoga Interventions for Chemotherapy-Induced Peripheral Neuropathy: Decision-Making to Enhance Protocol Quality Adherence | Integr Cancer Ther | Other/Mixed | 10.1177/15347354241261373 | Integr Cancer Ther. 2024 Jan-Dec;23:15347354241261373. doi: 10.1177/15347354241261373. |
| 40102693 | Sumner AL | 2025 | School-based yoga and mindfulness interventions for young adolescents: A qualitative study in a disadvantaged area | Br J Health Psychol | Healthcare | 10.1111/bjhp.12793 | Br J Health Psychol. 2025 May;30(2):e12793. doi: 10.1111/bjhp.12793. |
| 40669133 | Krupp K | 2025 | Policy implications of integrating yoga for healthy Aging: A stakeholder analysis in India | Geriatr Nurs | Other/Mixed | 10.1016/j.gerinurse.2025.103495 | Geriatr Nurs. 2025 Sep-Oct;65:103495. doi: 10.1016/j.gerinurse.2025.103495. Epub 2025 Jul 15. |
| 34469534 | Cattie JE | 2021 | Tailoring Trauma-Sensitive Yoga for High-Risk Populations in Public-Sector Settings | Int J Yoga Therap | Other/Mixed | 10.17761/2021-D-20-00035 | Int J Yoga Therap. 2021 Jan 1;31(1):Article_15. doi: 10.17761/2021-D-20-00035. |
| 39560022 | Rees T | 2024 | Yoga as an Adjunct Treatment to Manage Pain, Anxiety, Depression, and Stress During Hospital Stays: A Systematic Review | Int J Yoga Therap | Healthcare | 10.17761/2024-D-23-00047 | Int J Yoga Therap. 2024 Nov 1;34(2024):Article 15. doi: 10.17761/2024-D-23-00047. |
| 30958711 | Justice L | 2019 | Bridging Body and Mind: Case Series of a 10-Week Trauma-Informed Yoga Protocol for Veterans | Int J Yoga Therap | Healthcare | 10.17761/D-17-2019-00029 | Int J Yoga Therap. 2019 Nov;29(1):65-79. doi: 10.17761/D-17-2019-00029. Epub 2019 Apr 8. |
| 38102816 | Han S | 2023 | The Effects of Laughter Yoga on Perceived Stress, Positive Psychological Capital, and Exercise Capacity in Lung Cancer Chemotherapy Patients: A Pilot Randomized Trial | Integr Cancer Ther | Other/Mixed | 10.1177/15347354231218271 | Integr Cancer Ther. 2023 Jan-Dec;22:15347354231218271. doi: 10.1177/15347354231218271. |
| 30870013 | Rioux J | 2019 | Outcomes from a Whole-Systems Ayurvedic Medicine and Yoga Therapy Treatment for Obesity Pilot Study | J Altern Complement Med | Other/Mixed | 10.1089/acm.2018.0448 | J Altern Complement Med. 2019 Mar;25(S1):S124-S137. doi: 10.1089/acm.2018.0448. |
| 40467316 | Faravel K | 2025 | Impact of an educational physiotherapy-yoga intervention on perceived stress in women treated with brachytherapy for cervical cancer: a randomised controlled mixed study protocol (KYOCOL) | BMJ Open | Education | 10.1136/bmjopen-2024-098570 | BMJ Open. 2025 Jun 4;15(6):e098570. doi: 10.1136/bmjopen-2024-098570. |
| 37741064 | Trethewey E | 2023 | Yoga as an adjunct treatment for the eating disorders: A qualitive enquiry of clinician perspectives | Complement Ther Clin Pract | Healthcare | 10.1016/j.ctcp.2023.101796 | Complement Ther Clin Pract. 2023 Nov;53:101796. doi: 10.1016/j.ctcp.2023.101796. Epub 2023 Sep 10. |
| 33684325 | Sohl SJ | 2021 | Ensuring Yoga Intervention Fidelity in a Randomized Preference Trial for the Treatment of Worry in Older Adults | J Altern Complement Med | Other/Mixed | 10.1089/acm.2020.0476 | J Altern Complement Med. 2021 Jun;27(6):489-495. doi: 10.1089/acm.2020.0476. Epub 2021 Mar 8. |
| 33834599 | Capon H | 2021 | Yoga complements cognitive behaviour therapy as an adjunct treatment for anxiety and depression: Qualitative findings from a mixed-methods study | Psychol Psychother | Other/Mixed | 10.1111/papt.12343 | Psychol Psychother. 2021 Dec;94(4):1015-1035. doi: 10.1111/papt.12343. Epub 2021 Apr 8. |
| 36655641 | Borthakur D | 2023 | Yoga: A Natural Solution to Decrease Disease Burden in Children of MTHFR Deficient Parents | Clin Ter | Other/Mixed | 10.7417/CT.2023.5005 | Clin Ter. 2023 Jan-Feb;174(1):28-32. doi: 10.7417/CT.2023.5005. |
| 22894891 | Bryan S | 2012 | The effects of yoga on psychosocial variables and exercise adherence: a randomized, controlled pilot study | Altern Ther Health Med | Healthcare |  | Altern Ther Health Med. 2012 Sep-Oct;18(5):50-9. |
| 25747520 | Battle CL | 2015 | Potential for prenatal yoga to serve as an intervention to treat depression during pregnancy | Womens Health Issues | Other/Mixed | 10.1016/j.whi.2014.12.003 | Womens Health Issues. 2015 Mar-Apr;25(2):134-41. doi: 10.1016/j.whi.2014.12.003. |
| 25539839 | Vinchurkar SA | 2015 | Integrating yoga therapy in the management of urinary incontinence: a case report | J Evid Based Complementary Altern Med | Other/Mixed | 10.1177/2156587214563311 | J Evid Based Complementary Altern Med. 2015 Apr;20(2):154-6. doi: 10.1177/2156587214563311. Epub 2014 Dec 24. |
| 29096672 | Kwok JYY | 2017 | The effects of yoga versus stretching and resistance training exercises on psychological distress for people with mild-to-moderate Parkinson's disease: study protocol for a randomized controlled trial | Trials | Other/Mixed | 10.1186/s13063-017-2223-x | Trials. 2017 Nov 2;18(1):509. doi: 10.1186/s13063-017-2223-x. |
| 36633013 | Cartwright T | 2023 | 'It stretches your body but makes you feel good too': A qualitative study exploring young people's perceptions and experiences of yoga | J Health Psychol | Other/Mixed | 10.1177/13591053221146840 | J Health Psychol. 2023 Aug;28(9):789-803. doi: 10.1177/13591053221146840. Epub 2023 Jan 12. |
| 30653107 | Ramamoorthi R | 2019 | The effectiveness of yoga to prevent diabetes mellitus type 2: A protocol for systematic review and meta-analysis | Medicine (Baltimore) | Other/Mixed | 10.1097/MD.0000000000014019 | Medicine (Baltimore). 2019 Jan;98(3):e14019. doi: 10.1097/MD.0000000000014019. |
| 29131740 | Babbar S | 2017 | The Impact of Prenatal Yoga on Exercise Attitudes and Behavior: Teachable Moments from a Randomized Controlled Trial | Int J Yoga Therap | Other/Mixed | 10.17761/1531-2054-27.1.37 | Int J Yoga Therap. 2017 Nov;27(1):37-48. doi: 10.17761/1531-2054-27.1.37. |
| 40700530 | Bilagi A | 2025 | Health Disparities Induced by COVID-19 among Adolescents and Potential Mitigation through Yoga in the Post-Pandemic Era: A Narrative Review | Adv Mind Body Med | Other/Mixed |  | Adv Mind Body Med. 2025 Fall;39(4):20-25. |
| 40859498 | Li H | 2025 | Analysis of risk factors affecting knee injury during yoga based on medical big data analysis | Medicine (Baltimore) | Other/Mixed | 10.1097/MD.0000000000043926 | Medicine (Baltimore). 2025 Aug 22;104(34):e43926. doi: 10.1097/MD.0000000000043926. |
| 26283420 | Albert S | 2015 | Is 'mainstreaming AYUSH' the right policy for Meghalaya, northeast India? | BMC Complement Altern Med | Other/Mixed | 10.1186/s12906-015-0818-x | BMC Complement Altern Med. 2015 Aug 18;15:288. doi: 10.1186/s12906-015-0818-x. |
| 35833676 | Zaccari B | 2024 | Trauma Center Trauma-Sensitive Yoga Versus Cognitive Processing Therapy for Women Veterans With PTSD Who Experienced Military Sexual Trauma: A Feasibility Study | J Am Psychiatr Nurses Assoc | Healthcare | 10.1177/10783903221108765 | J Am Psychiatr Nurses Assoc. 2024 Apr;30(2):343-354. doi: 10.1177/10783903221108765. Epub 2022 Jul 14. |
| 25253113 | Subedi S | 2014 | Exploring different types of Hatha yoga for patients with cancer | Clin J Oncol Nurs | Other/Mixed | 10.1188/14.CJON.586-590 | Clin J Oncol Nurs. 2014 Oct;18(5):586-90. doi: 10.1188/14.CJON.586-590. |
| 38458849 | Hall S | 2024 | Implementation of a Clinic-Based Yoga Program for Chronic Pain | Pain Manag Nurs | Healthcare | 10.1016/j.pmn.2024.01.002 | Pain Manag Nurs. 2024 Jun;25(3):258-264. doi: 10.1016/j.pmn.2024.01.002. Epub 2024 Mar 7. |
| 40531549 | Liu H | 2025 | Predicting the influence of yoga on chronic venous insufficiency utilizing the Multi-Layer Perceptron Classifier | J Med Eng Technol | Other/Mixed | 10.1080/03091902.2025.2471331 | J Med Eng Technol. 2025 Jul;49(5):141-163. doi: 10.1080/03091902.2025.2471331. Epub 2025 Jun 18. |
| 40768382 | Özsezer G | 2025 | Real-Time Prediction of Correct Yoga Asanas in Healthy Individuals With Artificial Intelligence Techniques: A Systematic Review for Nursing | Nurs Open | Other/Mixed | 10.1002/nop2.70278 | Nurs Open. 2025 Aug;12(8):e70278. doi: 10.1002/nop2.70278. |
| 35736601 | Dossett A | 2022 | CE: An Evidence-Based Yoga Practice for Hospitalized Adults on Medical-Psychiatric Units | Am J Nurs | Healthcare | 10.1097/01.NAJ.0000840304.76501.96 | Am J Nurs. 2022 Jul 1;122(7):28-36. doi: 10.1097/01.NAJ.0000840304.76501.96. |
| 29131732 | Mason H | 2017 | Yoga and Healthcare in the United Kingdom | Int J Yoga Therap | Healthcare | 10.17761/1531-2054-27.1.121 | Int J Yoga Therap. 2017 Nov;27(1):121-126. doi: 10.17761/1531-2054-27.1.121. |
| 25617939 | Caffrey MK | 2014 | Evidence builds on yoga, but no reimbursement yet | Am J Manag Care | Other/Mixed |  | Am J Manag Care. 2014 May;20(8 Spec No.):E5. |
| 32182190 | Brennan MA | 2020 | Benefits of yoga in the treatment of eating disorders: Results of a randomized controlled trial | Eat Disord | Other/Mixed | 10.1080/10640266.2020.1731921 | Eat Disord. 2020 Jul-Aug;28(4):438-457. doi: 10.1080/10640266.2020.1731921. Epub 2020 Mar 17. |
| 24657958 | Tyagi A | 2014 | Yoga and hypertension: a systematic review | Altern Ther Health Med | Other/Mixed |  | Altern Ther Health Med. 2014 Mar-Apr;20(2):32-59. |
| 23070677 | Kusnick C | 2012 | Building bridges for yoga therapy research: the Aetna, Inc. mind-body pilot study on chronic and high stress | Int J Yoga Therap | Other/Mixed |  | Int J Yoga Therap. 2012;(22):91-2. |
| 39433109 | Phantayuth D | 2024 | Effectiveness of a 12-week combining tai chi and yoga program on pulmonary function and functional fitness in COPD patients | Respir Med | Other/Mixed | 10.1016/j.rmed.2024.107842 | Respir Med. 2024 Nov-Dec;234:107842. doi: 10.1016/j.rmed.2024.107842. Epub 2024 Oct 19. |
| 35315071 | O'Shea M | 2022 | Integration of hatha yoga and evidence-based psychological treatments for common mental disorders: An evidence map | J Clin Psychol | Other/Mixed | 10.1002/jclp.23338 | J Clin Psychol. 2022 Sep;78(9):1671-1711. doi: 10.1002/jclp.23338. Epub 2022 Mar 21. |
| 33652090 | Ciezar-Andersen SD | 2021 | A systematic review of yoga interventions for helping health professionals and students | Complement Ther Med | Education | 10.1016/j.ctim.2021.102704 | Complement Ther Med. 2021 May;58:102704. doi: 10.1016/j.ctim.2021.102704. Epub 2021 Feb 27. |
| 35732065 | Chaitanya Putchavayala K | 2022 | Development, Content Validation, and Feasibility of Yoga Module for Smartphone Addiction | Adv Mind Body Med | Healthcare |  | Adv Mind Body Med. 2022 Spring;36(2):14-22. |
| 41791008 | Mallaiah S | 2026 | Adapting Yoga Therapy to Meet the Needs of Inpatients Undergoing Hematopoietic Stem Cell Transplantation: Insights From an International, Multisite, Qualitative Study | JCO Glob Oncol | Healthcare | 10.1200/GO-25-00567 | JCO Glob Oncol. 2026 Mar;12(3):e2500567. doi: 10.1200/GO-25-00567. Epub 2026 Mar 6. |
| 28735778 | Groessl EJ | 2017 | Yoga for Military Veterans with Chronic Low Back Pain: A Randomized Clinical Trial | Am J Prev Med | Healthcare | 10.1016/j.amepre.2017.05.019 | Am J Prev Med. 2017 Nov;53(5):599-608. doi: 10.1016/j.amepre.2017.05.019. Epub 2017 Jul 20. |
| 39779637 | Chaudhary N | 2025 | Transcending Time and Space in the Search for Synthesis: A Commentary on Paranjpe's Understanding Yoga Psychology: Indigenous Knowledge with Global Relevance | Integr Psychol Behav Sci | Healthcare | 10.1007/s12124-024-09875-3 | Integr Psychol Behav Sci. 2025 Jan 9;59(1):15. doi: 10.1007/s12124-024-09875-3. |
| 34875056 | Owen-Smith A | 2021 | A Pilot Study to Adapt a Trauma-Informed, Mindfulness-Based Yoga Intervention for Justice-Involved Youth | Int J Yoga Therap | Justice/Carceral | 10.17761/2021-D-21-00032 | Int J Yoga Therap. 2021 Jan 1;31(1):Article_18. doi: 10.17761/2021-D-21-00032. |
| 22153143 | White LS | 2012 | Reducing stress in school-age girls through mindful yoga | J Pediatr Health Care | Education | 10.1016/j.pedhc.2011.01.002 | J Pediatr Health Care. 2012 Jan-Feb;26(1):45-56. doi: 10.1016/j.pedhc.2011.01.002. Epub 2011 Mar 2. |
| 40302463 | Solano-Durán P | 2025 | Trauma Center Trauma Sensitive Yoga Intervention (TCTSY). Improving Conditions for Incarcerated Women in Latin America | Med Sci Law | Justice/Carceral | 10.1177/00258024251329237 | Med Sci Law. 2025 Oct;65(4):339-344. doi: 10.1177/00258024251329237. Epub 2025 Apr 30. |
| 29741426 | Cramer H | 2018 | Yoga Therapy in the German Healthcare System | Int J Yoga Therap | Healthcare | 10.17761/2018-00006 | Int J Yoga Therap. 2018 Nov;28(1):133-135. doi: 10.17761/2018-00006. Epub 2018 May 9. |
| 37172418 | Smit C | 2023 | Recommending yoga for health: A survey of perceptions among healthcare practitioners in the UK | Complement Ther Clin Pract | Healthcare | 10.1016/j.ctcp.2023.101765 | Complement Ther Clin Pract. 2023 Aug;52:101765. doi: 10.1016/j.ctcp.2023.101765. Epub 2023 May 2. |
| 34144493 | Brosnan P | 2021 | Acceptability and feasibility of the online delivery of hatha yoga: A systematic review of the literature | Complement Ther Med | Other/Mixed | 10.1016/j.ctim.2021.102742 | Complement Ther Med. 2021 Aug;60:102742. doi: 10.1016/j.ctim.2021.102742. Epub 2021 Jun 15. |
| 39087486 | Groeger JL | 2025 | Yoga and Physical Therapy for Chronic Pain and Opioid Use Disorder Onsite in an Opioid Treatment Program: A Randomized Controlled Trial | Subst Use Addctn J | Healthcare | 10.1177/29767342241265929 | Subst Use Addctn J. 2025 Jan;46(1):175-183. doi: 10.1177/29767342241265929. Epub 2024 Aug 1. |
| 33965560 | Braun TD | 2021 | "We really need this": Trauma-informed yoga for Veteran women with a history of military sexual trauma | Complement Ther Med | Healthcare | 10.1016/j.ctim.2021.102729 | Complement Ther Med. 2021 Jun;59:102729. doi: 10.1016/j.ctim.2021.102729. Epub 2021 May 6. |
| 30948610 | Mathersul DC | 2019 | Study protocol for a non-inferiority randomised controlled trial of SKY breathing meditation versus cognitive processing therapy for PTSD among veterans | BMJ Open | Healthcare | 10.1136/bmjopen-2018-027150 | BMJ Open. 2019 Apr 3;9(4):e027150. doi: 10.1136/bmjopen-2018-027150. |
| 38345772 | Nilkantham S | 2023 | Knowledge, Attitude, and Practice of Yoga for Thyroid Dysfunction: A Cross-sectional Survey Using a Tableau Approach | Adv Mind Body Med | Other/Mixed |  | Adv Mind Body Med. 2023 Summer;37(3):23-32. |
| 32745069 | Salwa H | 2020 | Raising burden of non-communicable diseases: importance of integrating Yoga and Naturopathy at primary care level | J Complement Integr Med | Healthcare | 10.1515/jcim-2017-0019 | J Complement Integr Med. 2020 Aug 3;18(2):271-278. doi: 10.1515/jcim-2017-0019. |
| 22398354 | Bonura KB | 2011 | The psychological benefits of yoga practice for older adults: evidence and guidelines | Int J Yoga Therap | Other/Mixed |  | Int J Yoga Therap. 2011;(21):129-42. |
| 32608989 | Sharma KNS | 2020 | Integrated Yoga Practice in Cardiac Rehabilitation Program: A Randomized Control Trial | J Altern Complement Med | Healthcare | 10.1089/acm.2019.0250 | J Altern Complement Med. 2020 Oct;26(10):918-927. doi: 10.1089/acm.2019.0250. Epub 2020 Jun 29. |
| 33802581 | Heeter C | 2021 | Feasibility, Acceptability, and Outcomes of a Yoga-Based Meditation Intervention for Hospice Professionals to Combat Burnout | Int J Environ Res Public Health | Healthcare | 10.3390/ijerph18052515 | Int J Environ Res Public Health. 2021 Mar 3;18(5):2515. doi: 10.3390/ijerph18052515. |
| 27797663 | Ross A | 2016 | The Implementation of Patient-Reported Outcome Measures in Yoga Therapy | Int J Yoga Therap | Other/Mixed | 10.17761/1531-2054-26.1.113 | Int J Yoga Therap. 2016 Jan;26(1):113-121. doi: 10.17761/1531-2054-26.1.113. |
| 32503517 | Huberty J | 2020 | Online yoga to reduce post traumatic stress in women who have experienced stillbirth: a randomized control feasibility trial | BMC Complement Med Ther | Other/Mixed | 10.1186/s12906-020-02926-3 | BMC Complement Med Ther. 2020 Jun 5;20(1):173. doi: 10.1186/s12906-020-02926-3. |
| 40833192 | Allene C | 2025 | Yoga as a pre-treatment of EMDR to treat childhood abuse-related PTSD: feasibility and pilot study | Eur J Psychotraumatol | Other/Mixed | 10.1080/20008066.2025.2530917 | Eur J Psychotraumatol. 2025 Dec;16(1):2530917. doi: 10.1080/20008066.2025.2530917. Epub 2025 Aug 20. |
| 34185550 | Bringmann HC | 2021 | "To Be Finally at Peace with Myself": A Qualitative Study Reflecting Experiences of the Meditation-Based Lifestyle Modification Program in Mild-to-Moderate Depression | J Altern Complement Med | Other/Mixed | 10.1089/acm.2021.0038 | J Altern Complement Med. 2021 Sep;27(9):786-795. doi: 10.1089/acm.2021.0038. Epub 2021 Jun 29. |
| 32805735 | Moonaz S | 2021 | Characteristics of Clients Seeking Yoga Therapy in a University-Based Student Clinic | Int J Yoga Therap | Healthcare | 10.17761/2021-D-20-00003 | Int J Yoga Therap. 2021 Jan 1;31(1):Article_8. doi: 10.17761/2021-D-20-00003. |
| 36074961 | Di Mario S | 2023 | The Use of Yoga and Mindfulness-based Interventions to Reduce Stress and Burnout in Healthcare Workers: An Umbrella Review | Altern Ther Health Med | Healthcare |  | Altern Ther Health Med. 2023 Jan;29(1):29-35. |
| 40738219 | Loree AM | 2025 | Virtually-delivered prenatal yoga to prevent postpartum depression (PRYD) in women with a history of depression: Protocol for an exploratory pilot randomized controlled trial | Contemp Clin Trials | Other/Mixed | 10.1016/j.cct.2025.108032 | Contemp Clin Trials. 2025 Sep;156:108032. doi: 10.1016/j.cct.2025.108032. Epub 2025 Jul 28. |
| 25896571 | Sheffield KM | 2016 | Efficacy, Feasibility, and Acceptability of Perinatal Yoga on Women's Mental Health and Well-Being: A Systematic Literature Review | J Holist Nurs | Other/Mixed | 10.1177/0898010115577976 | J Holist Nurs. 2016 Mar;34(1):64-79. doi: 10.1177/0898010115577976. Epub 2015 Apr 20. |
| 29131735 | Taylor MJ | 2017 | Implementation of Yoga Therapy into U.S. Healthcare Systems | Int J Yoga Therap | Healthcare | 10.17761/1531-2054-27.1.115 | Int J Yoga Therap. 2017 Nov;27(1):115-119. doi: 10.17761/1531-2054-27.1.115. |
| 33741520 | Gautam S | 2021 | Yoga improves mitochondrial health and reduces severity of autoimmune inflammatory arthritis: A randomized controlled trial | Mitochondrion | Other/Mixed | 10.1016/j.mito.2021.03.004 | Mitochondrion. 2021 May;58:147-159. doi: 10.1016/j.mito.2021.03.004. Epub 2021 Mar 17. |
| 31895882 | Ratarasarn K | 2020 | Yoga and Tai Chi: a mind-body approach in managing respiratory symptoms in obstructive lung diseases | Curr Opin Pulm Med | Other/Mixed | 10.1097/MCP.0000000000000654 | Curr Opin Pulm Med. 2020 Mar;26(2):186-192. doi: 10.1097/MCP.0000000000000654. |
| 32074622 | Bali P | 2020 | Effectiveness of Yoga as the Public Health Intervention Module in the Management of Diabetes and Diabetes Associated Dementia in South East Asia: A Narrative Review | Neuroepidemiology | Other/Mixed | 10.1159/000505816 | Neuroepidemiology. 2020;54(4):287-303. doi: 10.1159/000505816. Epub 2020 Feb 19. |
| 27936953 | Donnelly KZ | 2017 | The feasibility and impact of a yoga pilot programme on the quality-of-life of adults with acquired brain injury | Brain Inj | Other/Mixed | 10.1080/02699052.2016.1225988 | Brain Inj. 2017;31(2):208-214. doi: 10.1080/02699052.2016.1225988. Epub 2016 Dec 12. |
| 40114123 | Risom SS | 2025 | The effect of MediYoga on sleep-quality, blood pressure and quality of life among older people with hypertension: study protocol of a pragmatic randomized controlled trial | BMC Complement Med Ther | Other/Mixed | 10.1186/s12906-025-04846-6 | BMC Complement Med Ther. 2025 Mar 20;25(1):109. doi: 10.1186/s12906-025-04846-6. |
| 38574083 | McCurdy BH | 2024 | Program evaluation of a school-based mental health and wellness curriculum featuring yoga and mindfulness | PLoS One | Healthcare | 10.1371/journal.pone.0301028 | PLoS One. 2024 Apr 4;19(4):e0301028. doi: 10.1371/journal.pone.0301028. eCollection 2024. |
| 33342752 | Moonaz S | 2022 | Yoga therapy DYADS: A novel approach to chronic pain management in underserved populations | Explore (NY) | Other/Mixed | 10.1016/j.explore.2020.12.002 | Explore (NY). 2022 Mar-Apr;18(2):195-199. doi: 10.1016/j.explore.2020.12.002. Epub 2020 Dec 13. |
| 26228429 | Pascoe MC | 2015 | A systematic review of randomised control trials on the effects of yoga on stress measures and mood | J Psychiatr Res | Other/Mixed | 10.1016/j.jpsychires.2015.07.013 | J Psychiatr Res. 2015 Sep;68:270-82. doi: 10.1016/j.jpsychires.2015.07.013. Epub 2015 Jul 13. |
| 34353794 | Moonaz S | 2021 | CLARIFY 2021: explanation and elaboration of the Delphi-based guidelines for the reporting of yoga research | BMJ Open | Other/Mixed | 10.1136/bmjopen-2020-045812 | BMJ Open. 2021 Aug 5;11(8):e045812. doi: 10.1136/bmjopen-2020-045812. |
| 37684609 | Miao C | 2023 | The effectiveness of mindfulness yoga on patients with major depressive disorder: a systematic review and meta-analysis of randomized controlled trials | BMC Complement Med Ther | Other/Mixed | 10.1186/s12906-023-04141-2 | BMC Complement Med Ther. 2023 Sep 8;23(1):313. doi: 10.1186/s12906-023-04141-2. |
| 38258889 | Krishnan A | 2024 | Integrative Treatment for Substance Use Disorders: Improving Outcomes Through Evidence-based Practice of Yoga-derived Breathwork and Meditation | J Addict Med | Other/Mixed | 10.1097/ADM.0000000000001263 | J Addict Med. 2024 Mar-Apr 01;18(2):103-109. doi: 10.1097/ADM.0000000000001263. Epub 2024 Jan 22. |
| 33373528 | Gorvine MM | 2021 | An Exploratory Study of the Acceptability and Feasibility of Yoga Among Women in Substance Use Disorder Recovery | J Altern Complement Med | Other/Mixed | 10.1089/acm.2020.0351 | J Altern Complement Med. 2021 Mar;27(3):273-281. doi: 10.1089/acm.2020.0351. Epub 2020 Dec 29. |
| 38013599 | Moonaz S | 2023 | Development and Implementation of a Flexible Yoga Therapy Protocol in the Group Acupuncture Therapy and Modified Yoga (GAPYOGA) Pilot Study | Int J Yoga Therap | Other/Mixed | 10.17761/2023-D-23-00011 | Int J Yoga Therap. 2023 Nov 1;33(2023):Article 13. doi: 10.17761/2023-D-23-00011. |
| 31377300 | Reindl D | 2020 | Elementary student and teacher perceptions of a mindfulness and yoga-based program in school: A qualitative evaluation | Explore (NY) | Healthcare | 10.1016/j.explore.2019.07.009 | Explore (NY). 2020 Mar-Apr;16(2):90-93. doi: 10.1016/j.explore.2019.07.009. Epub 2019 Jul 16. |
| 35055592 | Cagas JY | 2022 | For Exercise, Relaxation, or Spirituality: Exploring Participation Motives and Conformity to Masculine Norms among Male and Female Yoga Participants | Int J Environ Res Public Health | Other/Mixed | 10.3390/ijerph19020770 | Int J Environ Res Public Health. 2022 Jan 11;19(2):770. doi: 10.3390/ijerph19020770. |
| 34323666 | Dhungana RR | 2021 | Yoga for hypertensive patients: a study on barriers and facilitators of its implementation in primary care | Glob Health Action | Healthcare | 10.1080/16549716.2021.1952753 | Glob Health Action. 2021 Jan 1;14(1):1952753. doi: 10.1080/16549716.2021.1952753. |
| 26026151 | Brems C | 2015 | Improving access to yoga: barriers to and motivators for practice among health professions students | Adv Mind Body Med | Healthcare |  | Adv Mind Body Med. 2015 Summer;29(3):6-13. |
| 32160072 | Nicotera N | 2020 | The Influence of Trauma-Informed Yoga (TIY) on Emotion Regulation and Skilled Awareness in Sexual Assault Survivors | Int J Yoga Therap | Other/Mixed | 10.17761/2020-D-18-00031 | Int J Yoga Therap. 2020 Jan 1;30(1):19-31. doi: 10.17761/2020-D-18-00031. |
| 35910937 | Amitani M | 2022 | The Effect of Mindfulness Yoga in Children With School Refusal: A Study Protocol for an Exploratory, Cluster-Randomized, Open, Standard Care-Controlled, Multicenter Clinical Trial | Front Public Health | Healthcare | 10.3389/fpubh.2022.881303 | Front Public Health. 2022 Jul 13;10:881303. doi: 10.3389/fpubh.2022.881303. eCollection 2022. |
| 34331754 | de Barba YC | 2023 | Effects of Intuitive Eating on the Quality of Life and Wellbeing of Yoga Practitioners, Physical Activity Practitioners and Sedentary Individuals | Altern Ther Health Med | Other/Mixed |  | Altern Ther Health Med. 2023 May;29(4):82-91. |
| 40194465 | Karim FS | 2025 | Knowledge, attitude, practice, and perceived barriers to antenatal yoga among obstetricians and gynecologists in Bangladesh: A cross-sectional survey | Complement Ther Clin Pract | Other/Mixed | 10.1016/j.ctcp.2025.101981 | Complement Ther Clin Pract. 2025 May;59:101981. doi: 10.1016/j.ctcp.2025.101981. Epub 2025 Apr 3. |
| 36575507 | Courbet O | 2022 | Promoting psychological well-being in preschool children: study protocol for a randomized controlled trial of a mindfulness- and yoga-based socio-emotional learning intervention | Trials | Education | 10.1186/s13063-022-06979-2 | Trials. 2022 Dec 27;23(1):1050. doi: 10.1186/s13063-022-06979-2. |
| 33720749 | Anderson BJ | 2021 | Barriers and Facilitators to Implementing Bundled Acupuncture and Yoga Therapy to Treat Chronic Pain in Community Healthcare Settings: A Feasibility Pilot | J Altern Complement Med | Healthcare | 10.1089/acm.2020.0394 | J Altern Complement Med. 2021 Jun;27(6):496-505. doi: 10.1089/acm.2020.0394. Epub 2021 Mar 15. |
| 35301041 | O'Shea M | 2022 | A pragmatic preference trial of therapeutic yoga as an adjunct to group cognitive behaviour therapy versus group CBT alone for depression and anxiety | J Affect Disord | Other/Mixed | 10.1016/j.jad.2022.03.028 | J Affect Disord. 2022 Jun 15;307:1-10. doi: 10.1016/j.jad.2022.03.028. Epub 2022 Mar 15. |
| 38412056 | Wehrmann R | 2024 | Peer Support Groups Integrated with Trauma-Sensitive Yoga for Women Survivors of Sexual Violence: A Feasibility Study and Qualitative Examination | Int J Yoga Therap | Other/Mixed | 10.17761/2024-D-23-00026 | Int J Yoga Therap. 2024 Feb 1;34(2024):Article 1. doi: 10.17761/2024-D-23-00026. |
| 37461018 | Kwok JYY | 2023 | A randomized controlled trial on the effects and acceptability of individual mindfulness techniques - meditation and yoga - on anxiety and depression in people with Parkinson's disease: a study protocol | BMC Complement Med Ther | Other/Mixed | 10.1186/s12906-023-04049-x | BMC Complement Med Ther. 2023 Jul 17;23(1):241. doi: 10.1186/s12906-023-04049-x. |
| 28107843 | Waddington EA | 2017 | Staff perspectives regarding the implementation of a yoga intervention with chronic pain self-management in a clinical setting | Complement Ther Clin Pract | Healthcare | 10.1016/j.ctcp.2016.10.004 | Complement Ther Clin Pract. 2017 Feb;26:12-20. doi: 10.1016/j.ctcp.2016.10.004. Epub 2016 Nov 1. |
| 40265988 | Mona M | 2025 | Development, Validation, and Feasibility of Yoga Intervention Module for Optimizing Safe Use of Screen Time: A Pilot Study | Adv Mind Body Med | Healthcare |  | Adv Mind Body Med. 2025 Spring;39(2):9-16. |
| 41216743 | Pradhan N | 2025 | Understanding the feasibility of yoga and compassion meditation for stress management in mothers of children with intellectual disabilities in Nepal: a qualitative exploration | Glob Health Action | Other/Mixed | 10.1080/16549716.2025.2582260 | Glob Health Action. 2025 Dec;18(1):2582260. doi: 10.1080/16549716.2025.2582260. Epub 2025 Nov 11. |
| 33227624 | Law H | 2020 | Recruitment, retention, and adherence in a randomized feasibility trial of mindfulness-based stress reduction for patients with migraine | Complement Ther Med | Other/Mixed | 10.1016/j.ctim.2020.102610 | Complement Ther Med. 2020 Dec;55:102610. doi: 10.1016/j.ctim.2020.102610. Epub 2020 Nov 4. |
| 30712710 | Ponte SB | 2019 | Yoga in primary health care: A quasi-experimental study to access the effects on quality of life and psychological distress | Complement Ther Clin Pract | Other/Mixed | 10.1016/j.ctcp.2018.10.012 | Complement Ther Clin Pract. 2019 Feb;34:1-7. doi: 10.1016/j.ctcp.2018.10.012. Epub 2018 Oct 25. |
| 39253832 | Chopin SM | 2025 | Feasibility of a Virtual Adaptation of a Yoga Intervention for Veterans with Chronic Pain and Posttraumatic Stress Disorder | J Integr Complement Med | Healthcare | 10.1089/jicm.2024.0512 | J Integr Complement Med. 2025 Jan;31(1):92-99. doi: 10.1089/jicm.2024.0512. Epub 2024 Sep 10. |
| 24512477 | Hubbling A | 2014 | How mindfulness changed my sleep: focus groups with chronic insomnia patients | BMC Complement Altern Med | Other/Mixed | 10.1186/1472-6882-14-50 | BMC Complement Altern Med. 2014 Feb 10;14:50. doi: 10.1186/1472-6882-14-50. |
| 40830860 | H Yahara M | 2025 | Evaluation of the efficacy of aromatherapy and complementary yoga on indicators of medication regimen complexity: A retrospective study | BMC Complement Med Ther | Healthcare | 10.1186/s12906-025-05041-3 | BMC Complement Med Ther. 2025 Aug 19;25(1):310. doi: 10.1186/s12906-025-05041-3. |
| 22710259 | Slocum-Gori S | 2013 | Investigating the perceived feasibility of integrative medicine in a conventional oncology setting: yoga therapy as a treatment for breast cancer survivors | Integr Cancer Ther | Healthcare | 10.1177/1534735412443851 | Integr Cancer Ther. 2013 Mar;12(2):103-12. doi: 10.1177/1534735412443851. Epub 2012 Jun 17. |
| 28376861 | Schulz-Heik RJ | 2017 | Results from a clinical yoga program for veterans: yoga via telehealth provides comparable satisfaction and health improvements to in-person yoga | BMC Complement Altern Med | Healthcare | 10.1186/s12906-017-1705-4 | BMC Complement Altern Med. 2017 Apr 4;17(1):198. doi: 10.1186/s12906-017-1705-4. |
| 31464643 | Flehr A | 2019 | MindinBody - feasibility of vigorous exercise (Bikram yoga versus high intensity interval training) to improve persistent pain in women with a history of trauma: a pilot randomized control trial | BMC Complement Altern Med | Healthcare | 10.1186/s12906-019-2642-1 | BMC Complement Altern Med. 2019 Aug 29;19(1):234. doi: 10.1186/s12906-019-2642-1. |
| 21162384 | Lange B | 2010 | The yoga mat as common ground: policy from a holistic perspective | Beginnings | Other/Mixed |  | Beginnings. 2010 Fall;30(4):22-3. |
| 30477826 | Middleton KR | 2018 | Yoga for systemic lupus erythematosus (SLE): Clinician experiences and qualitative perspectives from students and yoga instructors living with SLE | Complement Ther Med | Healthcare | 10.1016/j.ctim.2018.09.001 | Complement Ther Med. 2018 Dec;41:111-117. doi: 10.1016/j.ctim.2018.09.001. Epub 2018 Sep 8. |
| 30055633 | Lopes CP | 2018 | Yoga and breathing technique training in patients with heart failure and preserved ejection fraction: study protocol for a randomized clinical trial | Trials | Healthcare | 10.1186/s13063-018-2802-5 | Trials. 2018 Jul 28;19(1):405. doi: 10.1186/s13063-018-2802-5. |
| 40045209 | Ward L | 2025 | Perceptions and experiences of chair-based yoga by older adults with multimorbidity - a qualitative process evaluation of the Gentle Years Yoga randomised controlled trial | BMC Geriatr | Healthcare | 10.1186/s12877-025-05782-3 | BMC Geriatr. 2025 Mar 5;25(1):152. doi: 10.1186/s12877-025-05782-3. |
| 32665041 | Lai KSP | 2020 | Breath Regulation and yogic Exercise An online Therapy for calm and Happiness (BREATH) for frontline hospital and long-term care home staff managing the COVID-19 pandemic: A structured summary of a study protocol for a feasibility study for a randomised controlled trial | Trials | Healthcare | 10.1186/s13063-020-04583-w | Trials. 2020 Jul 14;21(1):648. doi: 10.1186/s13063-020-04583-w. |
| 28675892 | Büssing A | 2018 | Treatment of Chronic Lower Back Pain: Study Protocol of a Comparative Effectiveness Study on Yoga, Eurythmy Therapy, and Physiotherapeutic Exercises | Complement Med Res | Other/Mixed | 10.1159/000471801 | Complement Med Res. 2018;25(1):24-29. doi: 10.1159/000471801. Epub 2017 Jul 3. |
| 41948021 | Zhou H | 2026 | Yoga and Tai chi: a cross-cultural comparative study of health benefits, cultural sustainability, and global public health implications | Front Public Health | Other/Mixed | 10.3389/fpubh.2026.1746662 | Front Public Health. 2026 Mar 23;14:1746662. doi: 10.3389/fpubh.2026.1746662. eCollection 2026. |
| 41225456 | Santarossa S | 2025 | Equity-focused barriers and facilitators to implementing a prenatal yoga intervention in a healthcare system: patient and provider perspectives | BMC Pregnancy Childbirth | Healthcare | 10.1186/s12884-025-08383-5 | BMC Pregnancy Childbirth. 2025 Nov 12;25(1):1197. doi: 10.1186/s12884-025-08383-5. |
| 41411986 | Algül G | 2026 | Integration of music, yoga and massage: Investigation of their effects on preschool children's social-emotional development and quality of life | J Pediatr Nurs | Education | 10.1016/j.pedn.2025.12.003 | J Pediatr Nurs. 2026 Jan-Feb;86:578-589. doi: 10.1016/j.pedn.2025.12.003. Epub 2025 Dec 17. |
| 40615420 | Irfan N | 2025 | Integrating AI predictive analytics with naturopathic and yoga-based interventions in a data-driven preventive model to improve maternal mental health and pregnancy outcomes | Sci Rep | Other/Mixed | 10.1038/s41598-025-07885-8 | Sci Rep. 2025 Jul 4;15(1):23878. doi: 10.1038/s41598-025-07885-8. |
| 41889607 | Tolbaños-Roche L | 2026 | Integrated comprehensive care in short-stay psychiatric units: a yoga-based adjunctive intervention and its association with wellbeing and patient satisfaction | Front Public Health | Healthcare | 10.3389/fpubh.2026.1772247 | Front Public Health. 2026 Mar 11;14:1772247. doi: 10.3389/fpubh.2026.1772247. eCollection 2026. |
| 41529878 | Shetty VV | 2026 | Exploring the mechanisms of yoga-based cardiac rehabilitation in heart failure via assessment of endothelial function, genomics and arterial health (Yoga-EndOmics): a study protocol | BMJ Open | Healthcare | 10.1136/bmjopen-2025-110239 | BMJ Open. 2026 Jan 13;16(1):e110239. doi: 10.1136/bmjopen-2025-110239. |
